# Identification of Rv3852 as an Agrimophol-Binding Protein in *Mycobacterium tuberculosis*

**DOI:** 10.1371/journal.pone.0126211

**Published:** 2015-05-15

**Authors:** Nan Zhao, Mingna Sun, Kristin Burns-Huang, Xiuju Jiang, Yan Ling, Crystal Darby, Sabine Ehrt, Gang Liu, Carl Nathan

**Affiliations:** 1 Department of Microbiology and Immunology, Weill Cornell Medical College, New York, New York, United States of America; 2 Tsinghua-Peking Center for Life Sciences and Department of Pharmacology and Pharmaceutical Sciences, School of Medicine, Tsinghua University, Beijing, P. R. China; University of Delhi, INDIA

## Abstract

*Mycobacterial tuberculosis (Mtb)* is able to preserve its intrabacterial pH (pH_IB_) near neutrality in the acidic phagosomes of immunologically activated macrophages and to cause lethal pathology in immunocompetent mice. In contrast, when its ability to maintain pH_IB_ homeostasis is genetically compromised, *Mtb* dies in acidic phagosomes and is attenuated in the mouse. Compounds that phenocopy the genetic disruption of *Mtb*’s pH_IB_ homeostasis could serve as starting points for drug development in their own right or through identification of their targets. A previously reported screen of a natural product library identified a phloroglucinol, agrimophol, that lowered *Mtb*’s pH_IB_ and killed *Mtb* at an acidic extrabacterial pH. Inability to identify agrimophol-resistant mutants of *Mtb* suggested that the compound may have more than one target. Given that polyphenolic compounds may undergo covalent reactions, we attempted an affinity-based method for target identification. The structure-activity relationship of synthetically tractable polyhydroxy diphenylmethane analogs with equivalent bioactivity informed the design of a bioactive agrimophol alkyne. After click-chemistry reaction with azido-biotin and capture on streptavidin, the biotinylated agrimophol analog pulled down the *Mtb* protein Rv3852, a predicted membrane protein that binds DNA *in vitro*. A ligand-protein interaction between agrimophol and recombinant Rv3852 was confirmed by isothermal calorimetry (ITC) and led to disruption of Rv3852’s DNA binding function. However, genetic deletion of rv3852 in *Mtb* did not phenocopy the effect of agrimophol on *Mtb*, perhaps because of redundancy of its function.

## Introduction

Tuberculosis (TB) caused by *Mycobacterium tuberculosis* (*Mtb*) remains the leading cause of death worldwide from a single bacterial infection [1]. Thus, it is an urgent task to develop drugs against TB with innovative mechanisms of action. One such mechanism could be disruption of *Mtb*’s homeostatic control of its intrabacterial pH (pH_IB_), as seen with genetic disruption of the gene encoding mycobacterial acid resistance protease (MarP) [2]. Natural products have been a prolific source of antibiotic chemophores [3]. Therefore, we screened a natural product library against *Mtb* that had been transformed with a ratiometric pH indicator and identified compounds that caused intrabacterial acidification of *Mtb* incubated at pH 4.5, as encountered in the phagosome of activated mouse macrophages [4, 5]. Agrimophol was the most potent compound identified in that screen to disrupt pH_IB_ homeostasis of *Mtb* and to kill *Mtb in vitro* [5]. Agrimophol is a phloroglucinol from *Agrimonia pilosa*, whose extracts have been used in traditional Chinese medicine to treat pulmonary infections.

We set out to identify agrimophol’s targets in *Mtb*. Agrimophol did not inhibit recombinant MarP (Fig. A in S1 File). Inability to identify agrimophol-resistant mutants encouraged us to use an affinity-based approach. As a precondition, a structure and activity relationship (SAR) analysis was conducted to identify bioactive analogs whose substituents could allow linkage to a solid phase support. This click-chemistry based approach succeeded in identifying an agrimophol binding protein, Rv3852. However, a contribution of Rv3852 to *Mtb*’s pH_IB_ homeostasis was not demonstrable.

## Materials and Methods

### Compounds

Natural agrimophol was extracted and purified from hairyvein agrinonia rhizome according to published procedures [5]. Agrimophol analogs a1, a2, a1b and a2b were synthesized following the synthetic route described in Fig. B in S1 File.

### Strains and media

The *Mtb* strain was H37Rv (ATCC 25618). *Bacille Calmette-Guérin* (*BCG*) (ATCC 35734) used in the pH_IB_ assay was transformed with a plasmid expressing a pH-sensitive ratiometric GFP (*BCG*-pHGFP). Both were cultivated in Difco Middlebrook 7H9 broth at pH 6.6 with 0.2% glycerol, 0.5% bovine serum albumin (BSA), 0.2% dextrose, 0.085% sodium chloride and 0.05% Tween 80 or on Difco 7H11 agar containing 0.5% glycerol and 10% OADC (oleic acid, albumin, dextrose and catalase supplement). Hygromycin (50 μg/mL), kanamycin (25 μg/mL) and zeocin (25 μg/mL) were added when needed. *Escherichia coli* (*E*. *coli*) strains DH5α and BL21(DE3) were used for amplification of plasmids and overexpression of recombinant Rv3852 respectively, while strains DB3.1 and Mach1 (Invitrogen) were used in construction of plasmids by a Gateway recombineering strategy. *E*. *coli* was cultured in LB broth or on LB agar with hygromycin (200 μg/mL), kanamycin (50 μg/mL), ampicillin (100 μg/mL), and chloramphenicol (25 μg/mL) and zeocin (25 μg/mL) as needed. Acidic buffer was 200 mM sodium phosphate and 100 mM citrate buffer at pH 4.5 with 0.02% tyloxapol (Pcit-Tyl-4.5). Lysis buffer was 50 mM monosodium phosphate, 300 mM sodium chloride and 10 mM imidazole, pH 8.0. Washing buffer was 50 mM monosodium phosphate, 300 mM and 20 mM imidazole, pH 8.0. Elution buffer was 50 mM monosodium phosphate, 300 mM sodium chloride and 250 mM imidazole, pH 8.0. Dialysis buffer was PBS containing 0.1% Triton-X100.

### pH_IB_ measurement and survival assays

Mid-log phase *BCG*-pHGFP was washed twice with Pcit-Tyl-4.5 and suspended in the same buffer to attain an OD_580_ of 0.2. Its pH_IB_ was detected after incubation with DMSO, 12.5 μM a1, a2 or 100 μM a1b, a2b at 37°C for 2 hours and 2 days. Stock concentrations of compounds in the test were 10 mM and final DMSO concentrations were 1% [5].

For survival assays, single cell suspensions of washed mid-log phase *BCG*-pHGFP, wild type *Mtb* or *rv3852* knockout *Mtb* were adjusted to an OD_580_ of 0.2 in Pcit-Tyl-4.5, treated as above for 2 and 6 days, serially diluted and plated on 7H11 plates with 50 μg/mL hygromycin. Colony forming units (CFU) were counted after two weeks [5].

### Identification of a1b-binding proteins in *BCG*

Mid-log phase *BCG* (250 mL) was washed with PBS and suspended in 1 mL PBS containing protease inhibitor cocktail (Roche) followed by bead beating 4 times. The cell lysate was ultracentrifuged at 414,630 g at 4°C for 1 hour to separate cytosolic (supernatant) and membrane-cell wall fractions (pellet). The pellet was washed 3 times with PBS, dissolved in PBS containing 1% Triton-X100 during rotation at 4°C for 1 hour and centrifuged at 414,630 g to provide a soluble membrane fraction (supernatant). Endogenous biotinylated and agarose-binding proteins in the soluble membrane fraction were removed by rotating with one-fifth volume of prewashed streptavidin agarose at 4°C for 1 hour. The protein concentration was finally adjusted to 1 mg/mL. The soluble membrane fraction was incubated with DMSO, 200 μM a1b or a2b at room temperature for 1 hour. Stock concentrations of compounds in the test were 10 mM and final DMSO concentrations were 2%. The samples were boiled in 4X SDS loading buffer at 95°C for 10 minutes, run on 12% SDS-PAGE and electroblotted to nitrocellulose membranes. The nitrocellulose membranes were treated with blocking buffer (Odyssey) at room temperature for 1 hour, exposed to a IRDye 800CW Streptavidin (Li-COR) at room temperature for 1 hour, washed with Tris-buffered saline with 0.05% Tween 20 (TBST) buffer 3 times (10 minutes each), and visualized with an infrared imaging system (Odyssey). One-fifth volume of prewashed streptavidin agarose was rotated with 500 μL of soluble membrane fraction samples that had been treated as above with DMSO, a1b or a2b at 4°C for 1 hours. Then the beads were washed three times by PBS, centrifuged at 13,000 g for 5 minutes, boiled in 4X SDS loading buffer at 95°C for 10 minutes and centrifuged again. The supernatant was run on SDS/PAGE. Excised lanes were treated with trypsin and the resulting peptides identified by MALDI-TOF MS.

### Preparation of recombinant Rv3852

According to the coding sequence of *rv3852* in *Mtb*, a forward primer CAACATATGCCAGACCCGCAGGATCGAC and a reverse primer CAACTCGAGCACTATGGTGCCAGCGCGTTC were synthesized (Invitrogen). NdeI and XhoI restriction sites are underlined. *rv3852* flanked with restriction sites was amplified from genomic DNA of *Mtb* using Phusion HF DNA Polymerase (New England BioLabs) and purified by QIAquick Gel Extraction Kit (Qiagen). The purified PCR product was subcloned into pET-28a(+) vector (Novagen) after digestion with NdeI and XhoI (New England BioLabs) and ligation with T4 DNA ligase (New England BioLabs). The resulting plasmid was transformed into chemically competent *E*. *coli* DH5α and plated on LB agar with 50 μg/mL kanamycin. Colonies were selected and expanded in LB broth with 50 μg/mL kanamycin. Amplified plasmids in *E*. *coli* DH5α were extracted by QIAprep Spin Miniprep Kit (Qiagen) and the DNA concentrations determined by spectrophotometer (NanoDrop). Cloned *rv3852* was sequenced (Macrogen) and then transform into chemically competent *E*. *coli* BL21(DE3), which was plated on LB agar with 50 μg/mL kanamycin. Colonies were expanded in LB broth containing 50 μg/mL kanamycin to mid-log phase. Expression was induced with 1 mM IPTG during rotation at 200 rpm at 25°C overnight. The cells were pelleted at 6000 g at 4°C for 20 minutes. The pellet was suspended in lysis buffer containing a protease inhibitor cocktail and lysed on ice by probe sonication four times for 30 seconds each with incubation on ice for 10 minutes between sonications. The lysate was separated into cytosolic and membrane-cell wall fractions by centrifuging at 16000 g at 4°C for 30 minutes. The membrane fraction in the pellet was dissolved in the lysis buffer containing protease inhibitor cocktail and 1% Triton-X100 by rotating at 4°C for 1 hour. After centrifuging at 16000 g at 4°C for 30 minutes once again, soluble membrane protein was obtained in the supernatant and applied to a column with prewashed nickel-nitrilotriacetic acid (Ni-NTA) agarose (Qiagen) at 4°C for 1 hour. Wash buffer was applied until no protein was detected in the eluate by Coomassie blue staining. Recombinant Rv3852 was eluted with elution buffer, concentrated on a 10 kD cut-off centrifugal filter unit (Millipore) and dialyzed in a cassette with 3.5 kD cut-off (Pierce). Purity was estimated after 12% SDS-PAGE by staining with Coomassie blue. The concentration was determined in DC Protein Assay (Bio-Rad). The molecular weight was determined by MALDI-TOF MS.

### Western blot, ITC and EMSA assays

Recombinant Rv3852 (100 ng) was incubated with DMSO, 1 μM a1b, a2b, a1, a2 or biotin at 37°C for 30 minutes. Stock concentrations of compounds in the test were 100 μM and final DMSO concentrations were 1%. Equimolar (160 ng) recombinant Rv2466c was used as a protein control. The samples were applied Western blot following the protocol described above.

For isothermal titration calorimetry (ITC), recombinant Rv3852 (217 μM) in dialysis buffer containing 0.6% DMSO and 300 μM agrimophol, a1 or a2 in the same dialysis buffer with 0.6% DMSO (stock concentrations of compounds in the test were 50 mM) were filtered and degassed by ultracentrifuging at 414,630 g at 4°C for 1 hour for use in a MicroCal Auto-iTC200 System (GE Healthcare). Each titration of recombinant Rv3852 against DMSO, a2, agrimophol or a1 involved 19 injections at a cell temperature of 25°C with reference power 10 μcal/second, initial delay of 60 seconds, stirring speed of 1000 rpm in an injection volume of 1 μL, with a duration of 4 seconds, spacing of 150 seconds and filter period of 5 seconds.

For electrophoretic mobility shift assay (EMSA), *proU* (MTB000030) in *Mtb* with extra 20 bases upstream and extra 189 bases downstream (proU2) was subcloned into pET-28a(+) vector following the procedures mentioned above [6]. Forward primer was CAAGAATTCGTAGGATCGCGAGGTCAG and reverse primer was CAAGAATTCCGGCGCCTTCCCGGGCCGGAAG. EcoRI restriction sites are underlined. Inserted proU2 was obtained by digesting the plasmid with EcoRI (New England BioLabs) and purifying by QIAquick Gel Extraction Kit. Recombinant Rv3852 (63, 125, 250, 500 and 1000 ng) was incubated with recombinant proU2 (500 ng) at room temperature for 30 minutes, run on the 1% agarose gel containing 0.01% ethidium bromide and visualized under UV light. Recombinant Rv2466c (1600 ng) was included as a protein control. For the competitive EMSA assay, 500 ng recombinant Rv3852 or 800 ng Rv2466c as a control were incubated with 25, 50 or 100 μM agrimophol, a1 or 100 μM a2 at room temperature for 1 hour before addition of 120 ng proU2 for another 30 min at room temperature. Stock concentrations of compounds in the test were 10 mM and final DMSO concentrations were 1%. The mixtures were electrophoresed on an agarose gel and observed under UV light.

### Construction of *rv3852* knockout *Mtb*

A plasmid with hygromycin resistance gene flanked with ~800 bp upstream and ~800 bp downstream of *rv3852* in genomic DNA of wild type *Mtb* were generated by using Gateway technology (Invitrogen) [7]. Replacement of *rv3852* with the hygromycin resistance gene was done by recombineering as previously described. On the generated plasmid, the recombineering PCR product, consisting of the hygromycin resistance cassette flanked with 500 bp upstream and 500 bp downstream of *rv3852* was amplified and purified. OD_600_ = 1.0 *Mtb* transformed with the plasmid expressing *Che9c* recombinase was induced by treating with 1 μM isovaleronitrile (IVN) at 37°C for 8 hours and then further treating with 2 M glycine at 37°C overnight. After induction, *Mtb* was converted to competent cell by washing it with 10% glycerol. Subsequently, 500 ng recombineering PCR product was transformed into 400 μL competent cells by electroporation (2.5 kV, 700 Ohm, 25 μF). The produced cells were incubated in 7H9 at 37°C overnight and then plated on 7H11 agar with 50 μg/mL hygromycin. Genomic DNA of hygromycin resistant *Mtb* was extracted for further verification by Southern blot and PCR [8]. For Southern blot analysis, genomic DNA from wild type *Mtb* and hygromycin resistant *Mtb* were digested by BclI (New England BioLabs) at 50°C overnight, run on the 1% agarose gel, transferred to the membrane (Amersham) and blotted by a designed probe. Hybridization and detection were carried out with an ECL direct nucleic acid labeling and detection system (GE Healthcare). For PCR analysis, primers were designed to confirm the allelic exchange and the presence of hygromycin resistance gene in the *rv3852* knockout *Mtb*. PCR products were then amplified and purified by using the designed primers on corresponding genomic DNA [8].

## Results

### Synthesis of agrimophol scaffold-hopping analogs

**Fig 1:**
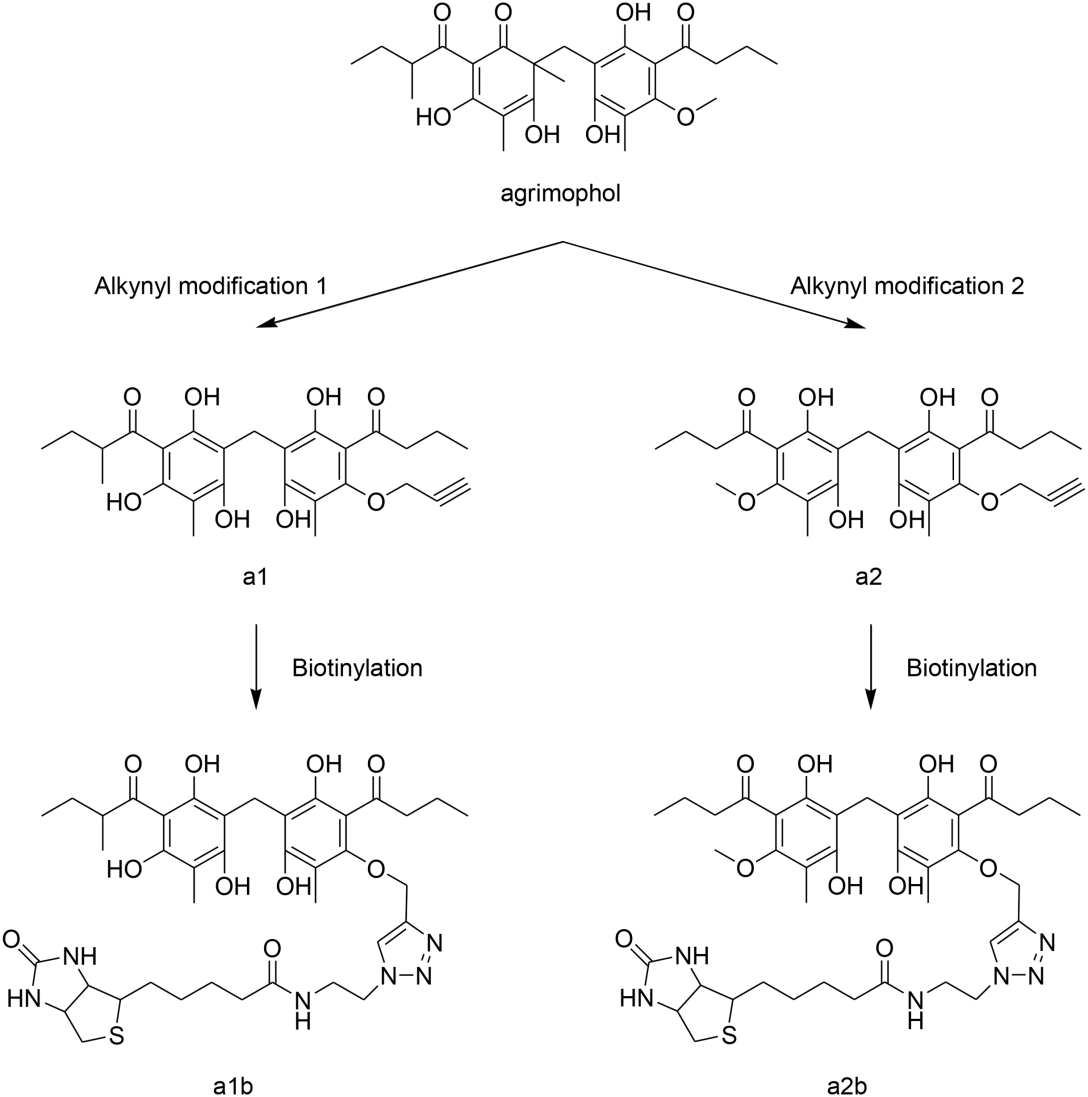
Synthesis of agrimophol analogs with alkyne group (a1 and a2) and corresponding biotinylated agrimophol analogs (a1b and a2b).

**Fig 2:**
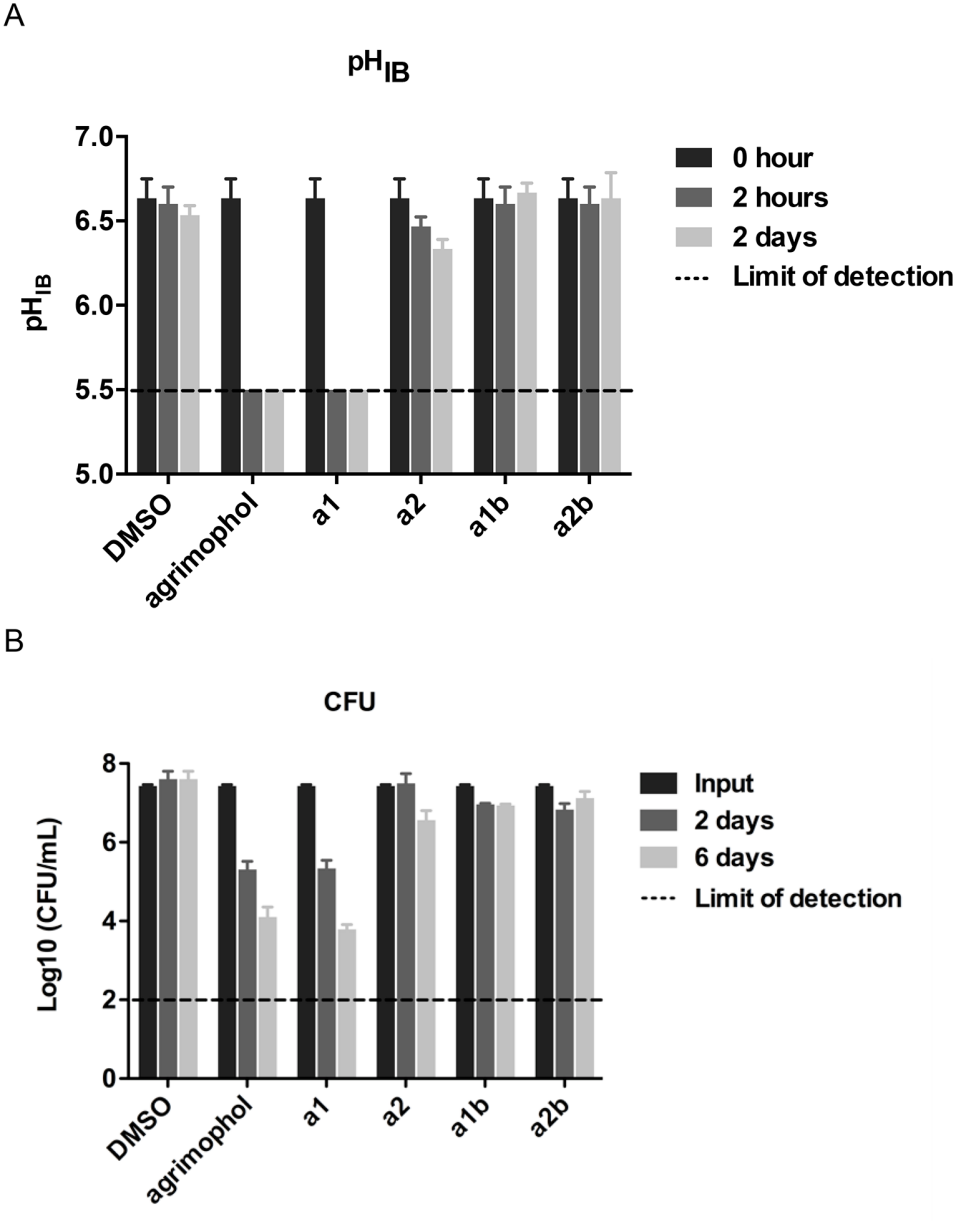
Activities of agrimophol and its analogs a1, a2, a1b and a2b on *BCG*. (A) pH_IB_ disruptive activity of agrimophol and its analogs a1 and a2 at 12.5 μM, a1b and a2b at 100 μM in Pcit-Tyl-4.5 at indicated time points. (B) CFU decreasing activity of agrimophol and its analogs a1 and a2 at 12.5 μM, a1b and a2b at 100 μM in Pcit-Tyl-4.5 at indicated time points. Means ± S. E. M. of triplicate samples represent three independent experiments. Some error bars are smaller than the symbols.

Because synthesis of natural agrimophol is difficult and the yields are very low, we took a scaffold-hopping approach to synthesize closely related, structurally simplified polyhydroxy diphenylmethanes (**a1** and **a2**) lacking one of agrimophol’s two chiral centers but retaining potency comparable to agrimophol’s in decreasing the pH_IB_ of *Mtb* incubated at acidic extracellular pH [9]. This allowed synthesis of a library of 104 analogs whose ability to disrupt pH_IB_ homeostasis was first tested in *BCG*, an attenuated vaccine strain of *Mycobacterium bovis*, then confirmed in *Mtb* (Sun M., et al., ms submitted). Based on this information, we synthesized two alkyne agrimophol analogs, **a1** and **a2**. As expected, **a1** remained bioactive at 12.5 μM, while the de-methylated, methoxy congener **a2** was inactive and served as a control (Figs 1, 2 and Fig. B in S1 File). The corresponding biotinylated triazoles generated through click chemistry, **a1b** and **a2b**, were both inactive up to the highest concentrations tested (100 μM) (Figs 1 and 2 and Fig. B in S1 File).

### Identification of an agrimophol target

**Fig 3:**
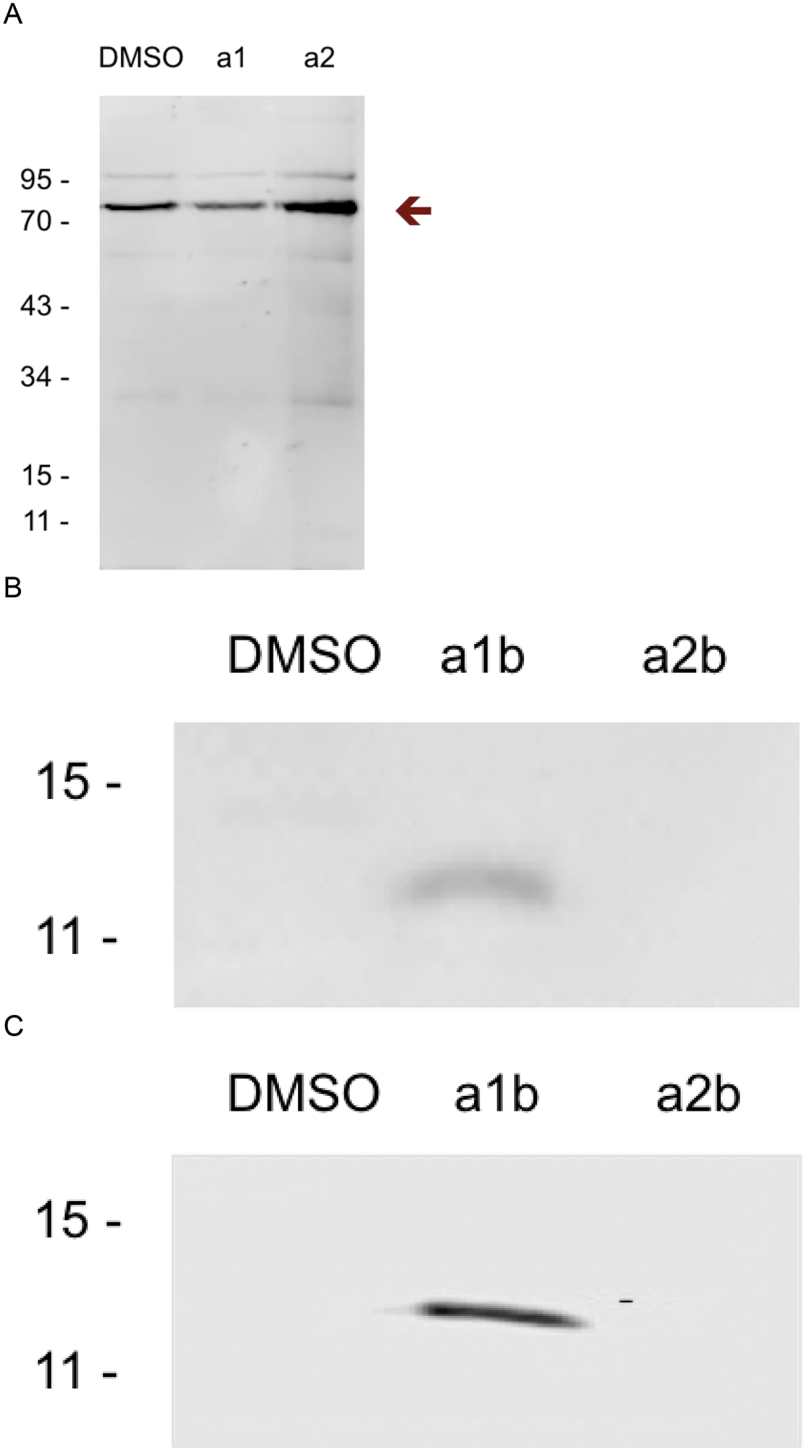
Identification and enrichment of a1b labeled proteins in the membrane-cell wall faction of *BCG* detecting by fluorescently tagged streptavidin in Western blot. (A) Only endogenous biotinylated proteins were detected in DMSO, a1b and a2b treated groups as indicated by arrow in CC-ABPP strategy. (B) A Mr 11–15 kD membrane protein was specifically detected in a1b treated group in ABPP strategy. (C) The Mr 11–15 kD membrane protein was enriched in a1b treated group by pulled down assay.

We initially applied click chemistry based activity-based protein profiling (CC-ABPP) by incubating a1 with *BCG*, separating the lysate into membrane-cell wall and cytosolic fractions, reacting the two fractions with azido-biotin, subjecting the mixtures to SDS-PAGE, transferring the proteins to nitrocellulose and detecting target proteins by blotting with fluorescein-tagged streptavidin [10]. The only proteins detected in either fraction were those that are endogenously biotinylated (Fig 3A; data in cytosolic fraction not shown) [11]. No a1 labeled proteins were detected. We thus switched to apply traditional ABPP by using the pre-biotinylated probe a1b [12]. A species migrating with apparent Mr 11–15 kD was then detected in the membrane-cell wall fraction after reaction with a1 but not DMSO or a2b (Fig 3B). After pull-down from a concentrated membrane-cell wall fraction, the protein was subjected to peptide mass fingerprinting (Fig 3C and Table 1) and identified as Mb3882 in *BCG*, whose homolog in *Mtb*, Rv3852, has the identical sequence [13, 14].

**Table 1:** Identification of Mb3882.

Protein	Molecular weight (D)	Unique peptides identified in the sequence
Mb3882	13814.70941	MPDPQDRPDSEPSDASTP*PAKK*L*PAKK*AA*KKAP*ARKT*PAKK*A*PAKK*TPAKGAK**SAPPKPAEAPVSLQQR**IETNGQLAAAAKDAAAQAK**STVEGANDALAR**NASVPAPSHSPVPLIVAVTLSLLALLLIRQLRRR

Detected sequences in MALDI-TOF MS are highlighted in bold. The 5 tetrapeptide repeats (4 PAKK and 1 KKAP) are italicized. Mb3882 was not identified in the DMSO treated group.

### Specific interaction of recombinant Rv3852 with agrimophol

**Fig 4:**
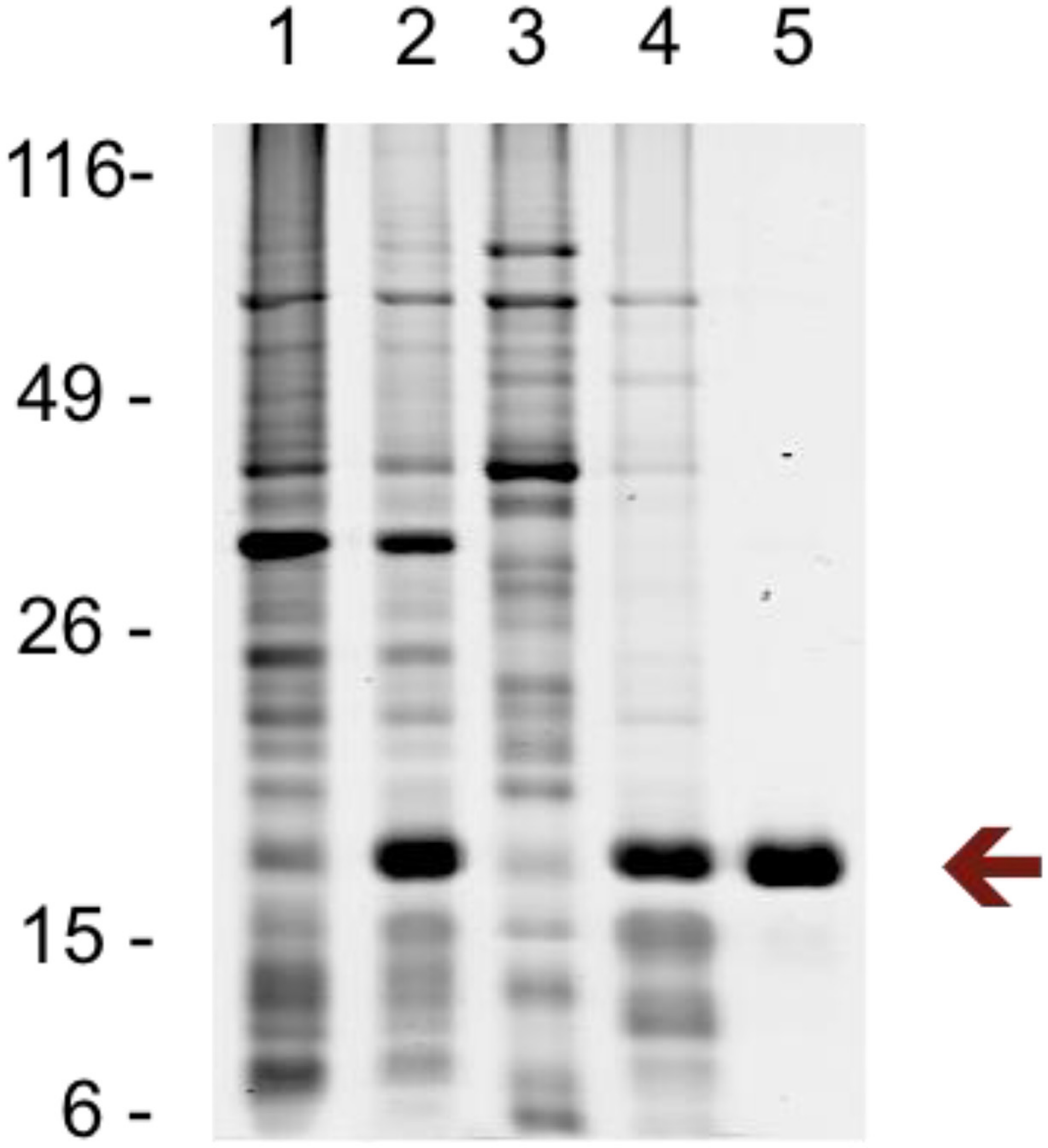
Overexpression and purification of Rv3852 in *E*. *coli*. Coomassie blue stained 12% SDS-PAGE. Lane 1: uninduced lysate; Lane 2: IPTG induced lysate; Lane 3: cytosolic fraction of IPTG induced lysate; Lane 4: membrane fraction of IPTG induced lysate (1% Triton-X100); Lane 5: purified Rv3852 (0.1% Triton-X100). Arrow indicates recombinant Rv3852.

We expressed Rv3852 with an N-terminal hexahistidine tag in *E*. *coli*, where it appeared chiefly in the membrane-cell wall fraction, and purified it by Ni-NTA chromatography (Fig 4) [15]. By MALDI-TOF MS, the Mr of purified recombinant Rv3852 was 15860.12, which is 127.28 kD less than calculated for the recombinant protein, probably reflecting truncation of the N-terminal methionine preceding the hexahistidine tag [16].

**Fig 5:**
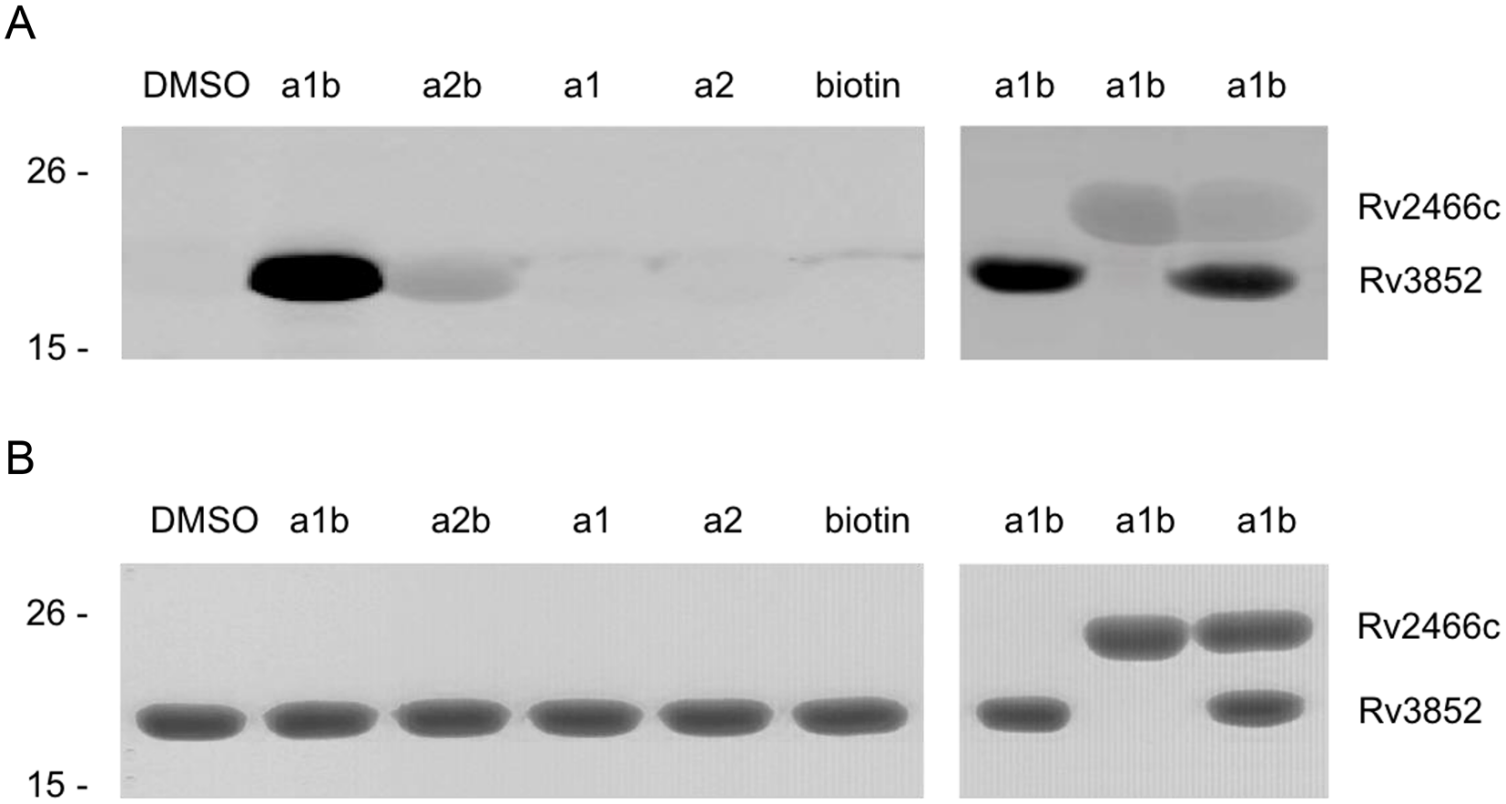
Specific binding between recombinant Rv3852 and a1b detected by fluorescently tagged streptavidin in Western blot. (A) Among DMSO, a1b, a2b, a1, a2 and biotin, only a1b demonstrated binding with recombinant Rv3852 as indicated in Western blot detecting by fluorescently tagged streptavidin. Rv2466c served as a control. (B) Equimolar recombinant Rv3852 (left) or Rv3852 and Rv2466c (right) were separated on 12% SDS-PAGE and stained by Coomassie Blue.

**Fig 6:**
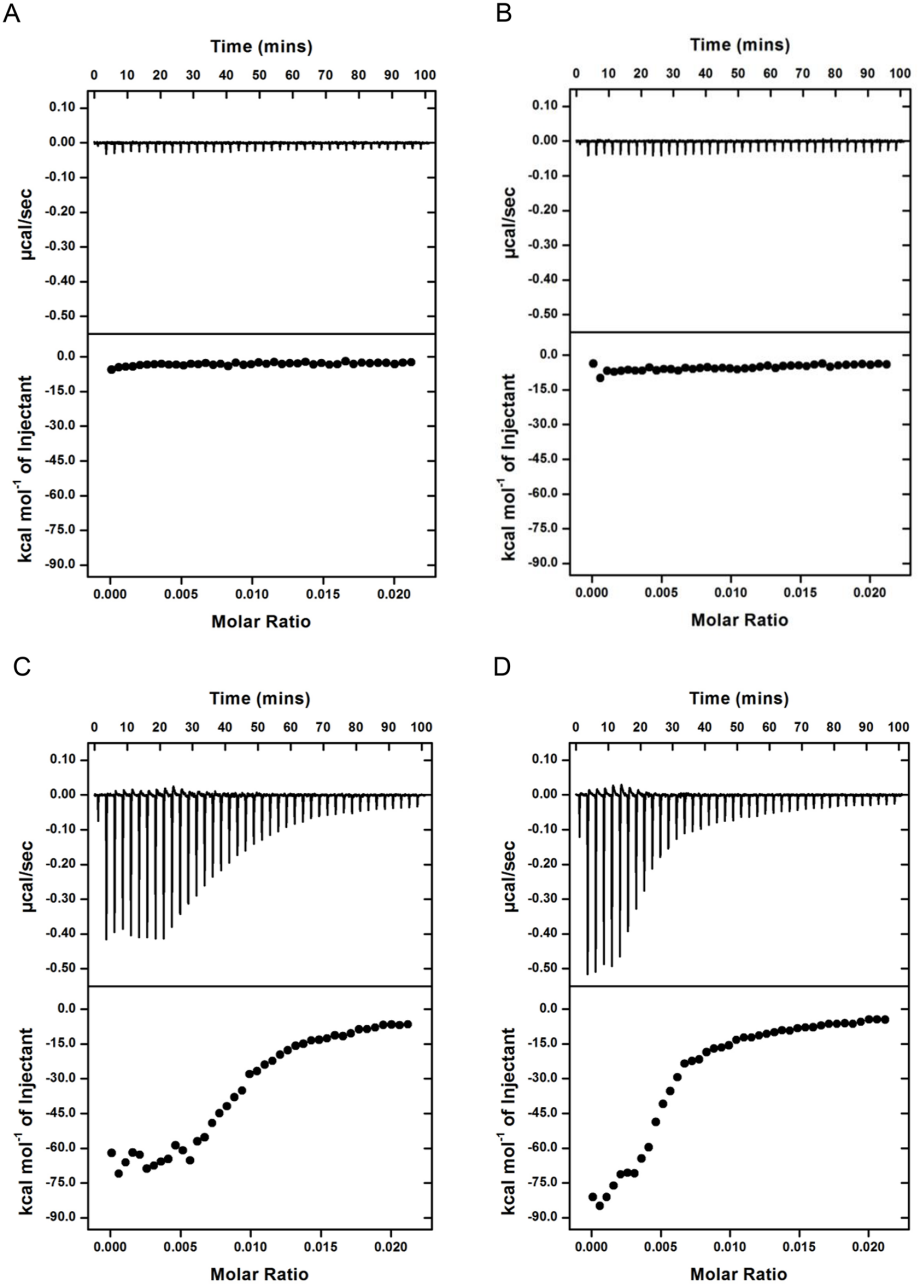
Specific binding between recombinant Rv3852 and agrimophol or a1 detected by ITC. Binding between recombinant Rv3852 with (A) DMSO, (B) a2, (C) agrimophol and (D) a1, respectively.

Western blot confirmed that recombinant Rv3852 specifically bound a1b (Fig 5). As chemical controls, DMSO, a1, a2 and biotin did not exhibit binding, whereas a2b demonstrated comparatively weak binding. As a protein control, recombinant Rv2466c only weakly interacted with a1b. Results were similar by ITC. DMSO and a2 did not demonstrate binding (Fig 6A and 6B), whereas agrimophol and a1 bound Rv3852 with stoichiometry of 10:1 (Fig 6C and 6D) and K_d_ of 34 μM and 22 μM, respectively.

### Functional impact of agrimophol on recombinant Rv3852

**Fig 7:**
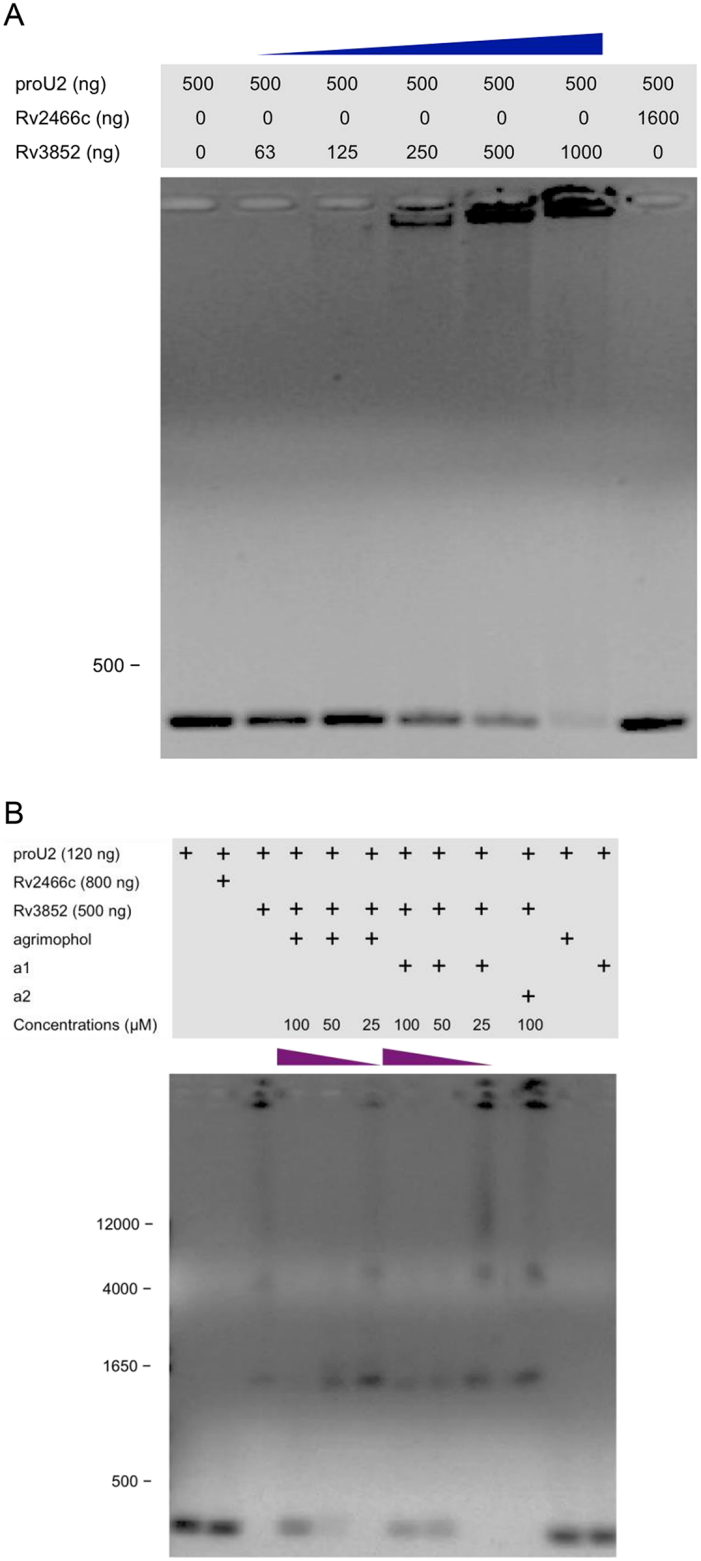
EMSA. (A) Recombinant Rv3852 abrogated the mobility of proU2 on 1% agarose gel. (B) agrimophol or a1 interfered with recombinant Rv3852’s mobility shift of proU2 on 1% agarose gel in a concentration-dependent manner.

The only known biochemical property of recombinant Rv3852 *in vitro* is its ability to bind DNA, although there is no evidence that this is a physiologic function [6, 15, 17]. To ascertain whether binding of agrimophol affected this function of Rv3852, we used an EMSA (Fig 7) with amplified and purified proU2, which is the *Mtb* tRNA gene *proU* (MTB000030) with a 20 bp 5’ extension and a 189 bp 3’ extension, as a source of DNA [6]. Pretreatment with recombinant Rv3852 impaired the mobility of proU2 in 1% agarose in a concentration-dependent manner, whereas recombinant Rv2466c used as a protein control had no such affect (Fig 7A) [6]. A competitive EMSA demonstrated that agrimophol and a1 could interfere with binding between recombinant Rv3852 and proU2 in a concentration-dependent manner (Fig 7B). In contrast, a2 did not exhibit any disruptive effect even at a higher concentration. In addition, mobility of proU2 was unaffected by agrimophol or a1 themselves in the absence of Rv3852.

### Lack of an acid-sensitive phenotype in *rv3852* knockout *Mtb*

**Fig 8:**
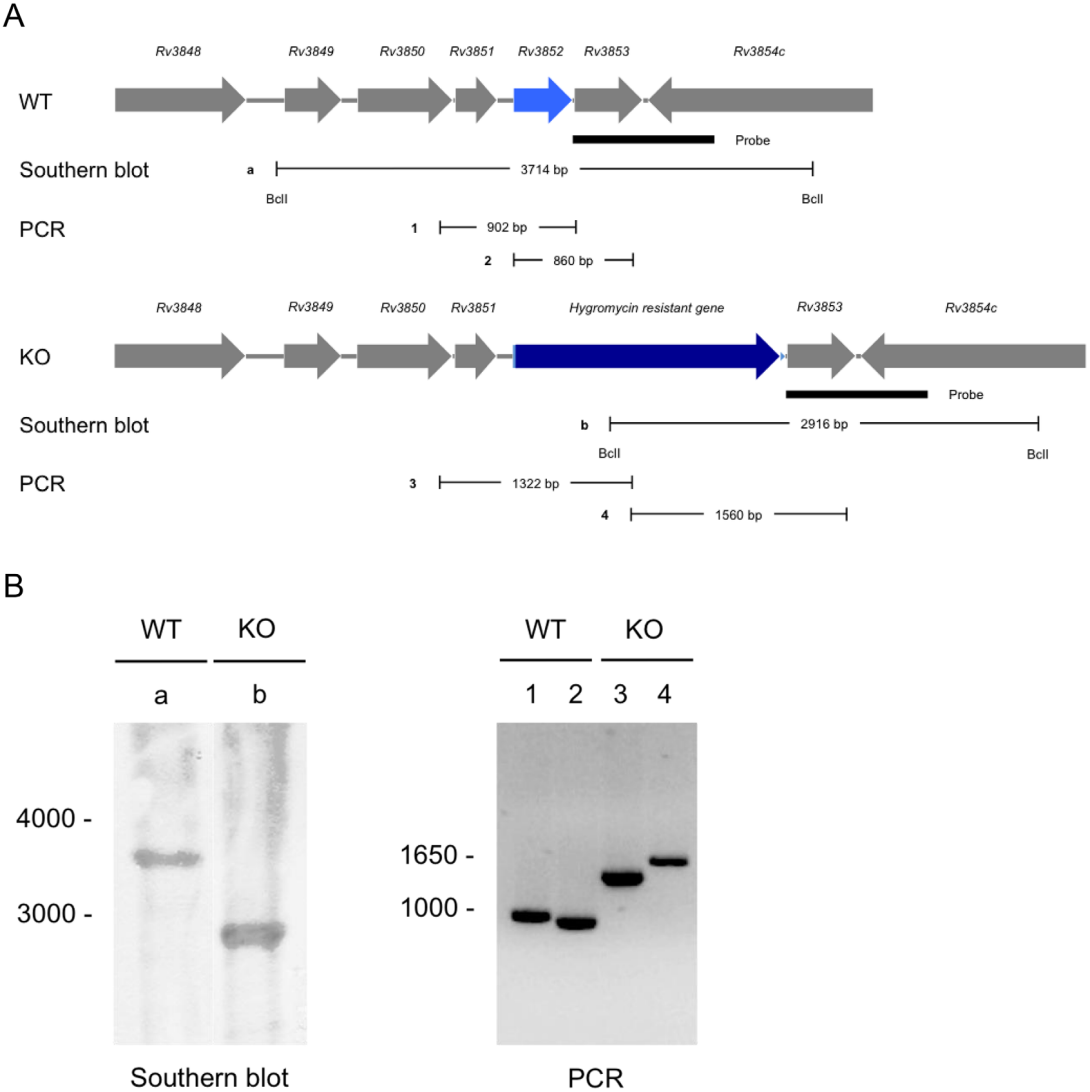
Construction of *rv3852* knockout *Mtb* and verification by Southern blot and PCR. (A) Upper panel displays the genetic organization of the *rv3852* region in *Mtb* (WT), Lower panel displays the same region with replacement of *rv3852* by hygromycin resistance gene in *rv3852* knockout *Mtb* (KO). Filled rectangle indicates the location of probe used in the Southern blot. Sites of digestion by BclI on genomic DNA from WT and KO as well as the sizes of the generated DNA fragment (a and b) are demonstrated under each panel. PCR products from genomic DNA from WT (1 and 2) and KO (3 and 4) are denoted. (B) Left, Southern blot of BclI digested genomic DNA from WT and KO. Calculated sizes of the fragments hybridizing with the probe were 3714 bp (WT) and 2916 bp (KO) as indicated in A. Right, PCR products from genomic DNA from WT and KO. The calculated sizes of the PCR products were 902 (Lane 1), 860 (Lane 2) for WT and 1322 (Lane 3), 1560 bp (Lane 4) for KO as indicated in A.

**Fig 9:**
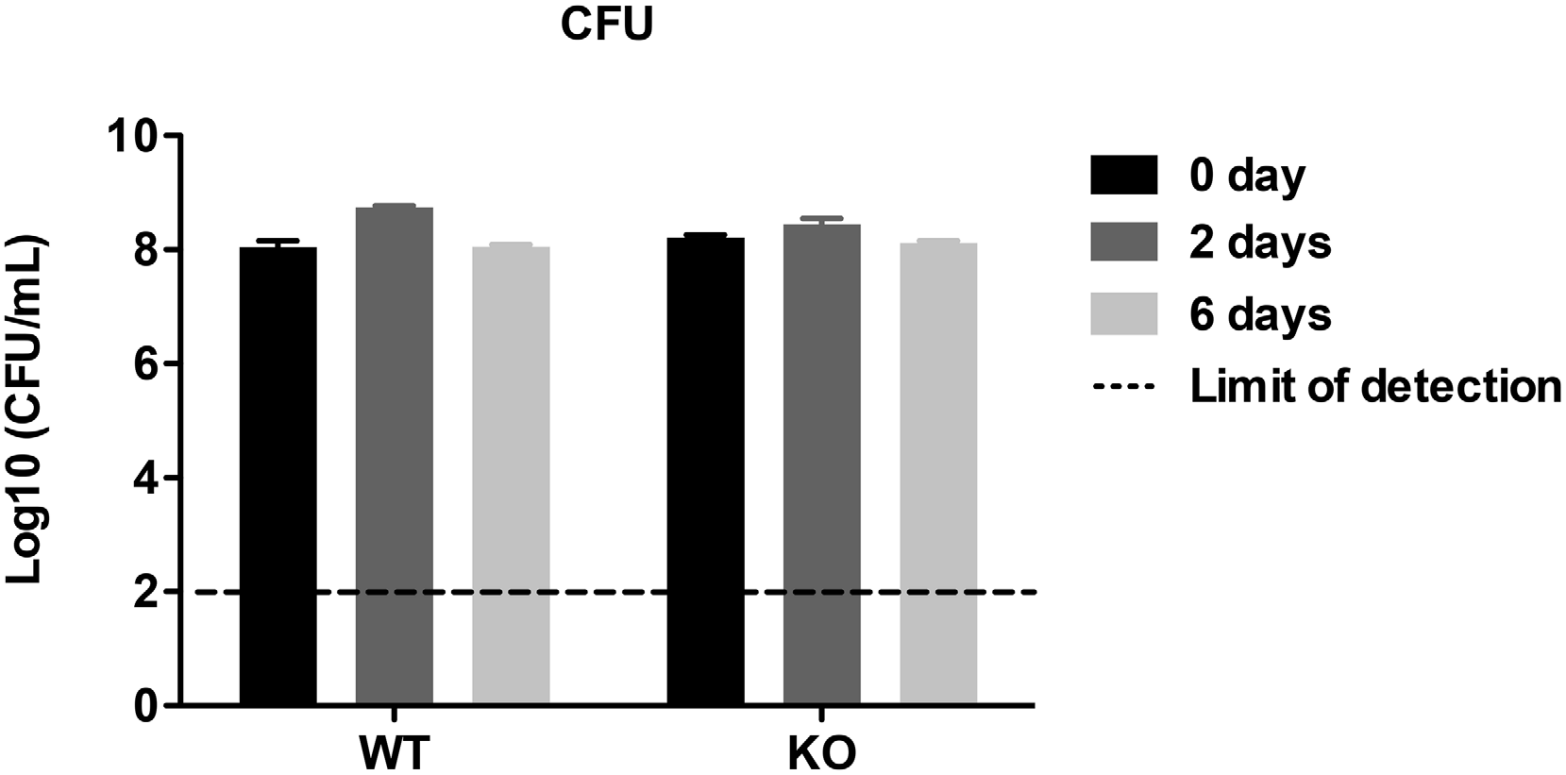
Survival of WT and KO in Pcit-Tyl-4.5 at indicated time points. Means ± S. E. M. of triplicate samples represent three independent experiments. Some error bars are smaller than the symbols.

To test if Rv3852 deficiency would phenocopy agrimophol treatment, we deleted *rv3852* in *Mtb* through homologous recombination and verified the deletion by Southern blot and PCR (Fig 8) [7, 8]. Comparison of the survival of wild type *Mtb* and the *rv3852* knockout *Mtb* in Pcit-Tyl-4.5 did not demonstrate a difference; both strains maintained the same level of CFU over the 6 days period of observation (Fig 9) [2].

## Discussion

In seeking the functionally relevant targets of agrimophol as a disruptor of *Mtb’s* pH_IB_ homeostasis, we focused on membrane proteins as a likely site for regulation of the transmembrane pH gradient. Membrane proteins are a main class of targets of chemical inhibitors, drug candidates and drugs [18].

In this study, CC-ABPP was ineffective, while use of the pre-clicked probe a1b identified an agrimophol binding protein (Fig 3). We suspect the lack of efficacy of CC-ABPP may have been due to chelation of the copper catalyst by multiple hydroxyl groups on a1, reducing the efficiency of the click reaction and/or triggering production of radicals that could impair binding between a1 and its targets [19, 20]. Alternatively, aggregation of the polyphenol may have sterically hindered contact between the alkyne group and the azide group [21].

Rv3852 is a protein of unknown function that is predicted by hidden Markov models (HMM) to cross the cell membrane [22]. Consistent with this, Rv3852 was identified as a membrane-associated protein in Triton X-114 extracts of *Mtb*, was identified in the *BCG* membrane-cell wall fraction in the present study and appeared in the membrane-cell wall fraction of *E*. *coli* when over-expressed as a recombinant protein (Fig 4) [23]. Thus, its reported ability to bind DNA, confirmed here, is of uncertain physiologic relevance (Fig 7) [6, 15, 17]. Nonetheless, the ability of agrimophol to interfere with the DNA binding ability of recombinant Rv3852 *in vitro* added further evidence to the pull-down and ITC studies that agrimophol binds Rv3852 directly. Controls with a1, a2, a2b, biotin and Rv2466c argued for the specificity of the interaction, and ITC indicated that the binding had a K_d_ of 34 μM, although ITC did not distinguish whether the binding was noncovalent or covalent and reversible.

The molar ratio of binding between recombinant Rv3852 and agrimophol or a1 as inferred from ITC was 1:10. This may reflect oligomerization of recombinant Rv3852 in aqueous solution, a non-native environment for this membrane-associated protein. It was reported that recombinant Rv3852 existed in solution as a dimer and formed higher oligomers when bound to DNA, as indicated by surface plasmon resonance (SPR), where the stoichiometry between DNA and monomeric recombinant Rv3852 was determined to be 1:10 [6]. In our ITC experiments, agrimophol or a1 may have played the same inducing role as DNA played in SPR.

The 5 lysine-rich tetrapeptide repeats (4 PAKK and 1 KKAP) in the N-terminal region of recombinant Rv3852 were able to bind DNA with high affinity (Fig 7 and Table 1) [24]. Additionally, lysines in proteins can react with natural polyphenols [25, 26]. Therefore, agrimophol and a1 may disrupt the binding between recombinant Rv3852 and proU2 DNA through interacting with the tetrapeptide repeats (Fig 7).

Microarray analysis was used to monitor the transcriptional response of *Mtb* to starvation in PBS for 4 h, 24 h and 96 h [27]. At 4 hours, levels of *rv3852* mRNA were upregulated 2.49-fold. Two other mycobacterial DNA binding proteins with repetitive PAKKs at the C-terminal region, Mb3010c (a histone-like protein in *BCG*, the homolog of Rv2986c in *Mtb*) and Rv0475 (heparin binding hemagglutinin, hbha, in *Mtb*) were required for resistance of mycobacteria to acidic stress *in vitro* [28, 29]. All 3 mycobacterial DNA binding proteins may help *Mtb* resist certain stresses. Perhaps they do so redundantly. Redundancy of function could explain the lack of an acid-sensitivity phenotype in *rv3852* knockout *Mtb*, a hypothesis consistent with our inability to isolate an agrimophol-resistant mutant. Perhaps agrimophol disrupts the function both of Rv3852 and of another protein or proteins that share functions with Rv3852, but the levels of the other protein(s) were too low to be detected by the pull-down method used here.

## Conclusions

Agrimophol is a natural product that interferes with pH_IB_ homeostasis of *Mtb*. We took advantage of knowledge of the structure-activity relationship of a closely related compound series to design a probe with which to affinity-purify agrimophol targets. This approach identified Rv3852, a membrane-anchored yet histone-like protein. ITC and EMSA confirmed a specific interaction of agrimophol with recombinant Rv3852. However, in contrast to treatment with agrimophol, deletion of *rv3852* did not compromise *Mtb’s* survival at pH’s found in macrophage phagosomes. This may reflect functional redundancy of Rv3852 among agrimophol’s targets.

## Supporting Information

S1 File This file contains the following. Fig. A Agrimophol does not inhibit MarP; Fig. B Synthetic route of a1, a2, a1b and a2b; Fig. C ^1^H NMR (300 MHz, CDCl_3_) of a1; Fig. D ^13^C NMR (150 MHz, CDCl_3_) of a1; Fig. E HRMS of a1; Fig. F ^1^H NMR (300 MHz, CDCl_3_) of a2; Fig. G ^13^C NMR (75 MHz, CDCl_3_) of a2; Fig. H HRMS of a2; Fig. I ^1^H NMR (300 MHz, DMSO-*d*_*6*_) of a1b; Fig. J ^13^C NMR (125 MHz, DMSO-*d*_*6*_) of a1b; Fig. K HRMS of a1b; Fig. L ^1^H NMR (300 MHz, DMSO-*d*_*6*_) of a2b; Fig. M ^13^C NMR (125 MHz, DMSO-*d*_*6*_) of a2b; Fig. N HRMS of a2b; Synthetic methods; References.
(DOCX)
