# Correction: Identification of Rv3852 as an Agrimophol-Binding Protein in *Mycobacterium tuberculosis*

**DOI:** 10.1371/journal.pone.0131145

**Published:** 2015-06-17

**Authors:** The PLOS ONE Staff

S1 File contains tracked changes that were made during the preparation of the manuscript for production. The publisher apologizes for the error. Please view the correct S1 File below.

## Supporting Information

S1 File This file contains the following. Fig. A Agrimophol does not inhibit MarP; Fig. B Synthetic route of a1, a2, a1b and a2b; Fig. C^1^H NMR (300 MHz, CDCl_3_) of a1; Fig. D ^13^C NMR (150 MHz, CDCl_3_) of a1; Fig. E HRMS of a1; Fig. F ^1^H NMR (300 MHz, CDCl_3_) of a2; Fig. G ^13^C NMR (75 MHz, CDCl_3_) of a2; Fig. H HRMS of a2; Fig. I ^1^H NMR (300 MHz, DMSO-*d*_*6*_) of a1b; Fig. J ^13^C NMR (125 MHz, DMSO-*d*_*6*_) of a1b; Fig. K HRMS of a1b; Fig. L ^1^H NMR (300 MHz, DMSO-*d*_*6*_) of a2b; Fig. M ^13^C NMR (125 MHz, DMSO-*d*_*6*_) of a2b; Fig. N HRMS of a2b; Synthetic methods; References.
(DOCX)
