# Supplementary material for: Identification of Rv3852 as an Agrimophol-Binding Protein in Mycobacterium tuberculosis
Source: PLoS One. 2015 May 15;10(5):e0126211. doi: 10.1371/journal.pone.0126211 (PMC4433263; doi:10.1371/journal.pone.0126211)
Supplement: S1 File — Fig. A Agrimophol does not inhibit MarP; Fig. B Synthetic route of a1, a2, a1b and a2b; Fig. C 1H NMR (300 MHz, CDCl3) of a1; Fig. D 13C NMR (150 MHz, CDCl3) of a1; Fig. E HRMS of a1; Fig. F 1H NMR (300 MHz, CDCl3) of a2; Fig. G 13C NMR (75 MHz, CDCl3) of a2; Fig. H HRMS of a2; Fig. I 1H NMR (300 MHz, DMSO-d 6) of a1b; Fig. J 13C NMR (125 MHz, DMSO-d 6) of a1b; Fig. K HRMS of a1b; Fig. L 1H NMR (300 MHz, DMSO-d 6) of a2b; Fig. M 13C NMR (125 MHz, DMSO-d 6) of a2b; Fig. N HRMS of a2b; Synthetic methods; References. (DOCX) [file pone.0126211.s001.docx]

**Identification of Rv3852 as an Agrimophol-Binding Protein in *Mycobacterium tuberculosis***

# Nan Zhao^1¶^, Mingna Sun^2¶^, Kristin Burns-Huang^1^, Xiuju Jiang^1^, Yan Ling^1^, Crystal Darby^1^, Sabine Ehrt^1^, Gang Liu^2^* and Carl Nathan^1^*

1 Department of Microbiology and Immunology, Weill Cornell Medical College, New York, New York, United States of America

2 Tsinghua-Peking Center for Life Sciences and Department of Pharmacology and Pharmaceutical Sciences, School of Medicine, Tsinghua University, Beijing, P. R. China

^¶^ These authors contributed equally to this work.

* Corresponding authors

Emails: gangliu27@biomed.tsinghua.edu.cn (GL) or cnathan@med.cornell.edu (CN).

**SI File Supporting Information**

**Contents**

**Fig. A** Agrimophol does not inhibit MarP

**Fig. B** Synthetic route of a1, a2, a1b and a2b

**Fig. C** ^1^H NMR (300 MHz, CDCl_3_) of a1

**Fig. D** ^13^C NMR (150 MHz, CDCl_3_) of a1

**Fig. E** HRMS of a1

**Fig. F** ^1^H NMR (300 MHz, CDCl_3_) of a2

**Fig. G** ^13^C NMR (75 MHz, CDCl_3_) of a2

**Fig. H** HRMS of a2

**Fig. I** ^1^H NMR (300 MHz, DMSO*-d_6_*) of a1b

**Fig. J** ^13^C NMR (125 MHz, DMSO*-d_6_*) of a1b

**Fig. K** HRMS of a1b

**Fig. L** ^1^H NMR (300 MHz, DMSO*-d_6_*) of a2b

**Fig. M** ^13^C NMR (125 MHz, DMSO*-d_6_*) of a2b

**Fig. N** HRMS of a2b

**Synthetic methods**

**References**

**S1 Fig. Agrimophol does not inhibit MarP.** One μM recombinant MarP was combined with 7 μM β-casein and 20 μM FP-TAMRA or agrimophol in Tris-HCl buffer at pH 7.4. The reaction was incubated at room temperature for 15 minutes, then at 37°C overnight. Digestions were detected by running SDS-PAGE and staining gel by Coomassie Blue.

**
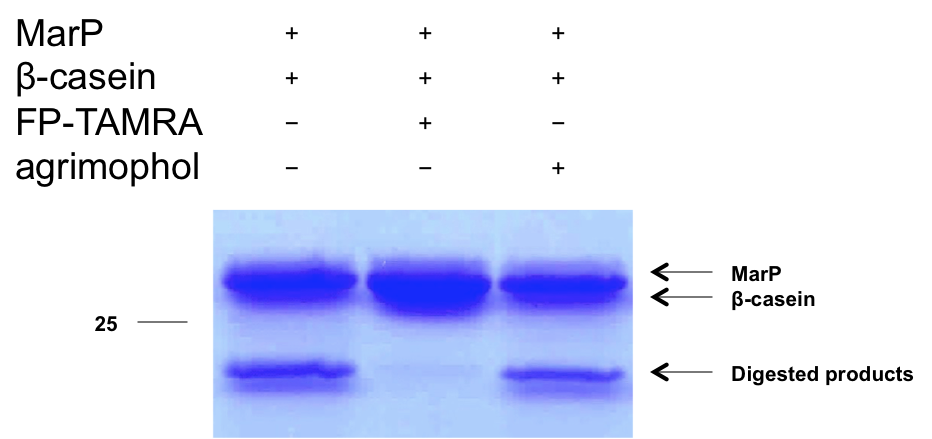
**

**S2 Fig. Synthetic route of a1, a2, a1b and a2b.** Reagents and conditions: (A) POCl_3_, DMF, room temperature, 2 hours; (B) NaBH_3_CN, HCl, THF, room temperature, 2 hours; (C) Butyryl chloride or 2-methylbutyryl chloride, AlCl_3_, 1,2-dichloroethane, from room temperature to 50 °C; (D) MOMCl, K_2_CO_3_, acetone, room temperature; (E) Me_2_SO_4_, K_2_CO_3_, acetone, reflux; (F) Propargyl alcohol, PPh_3_, DEAD, THF, room temperature; (G) Concentrated HCl, MeOH, reflux; (H) HCHO, acetic acid, room temperature; (I) Biotinylated azide, CuSO_4_^.^5H_2_O, sodium ascorbate, *t*-BuOH/H_2_O (1:1), 45 °C, 12 hours.

**S3 Fig. ^1^H NMR (300 MHz, CDCl_3_) of a1**


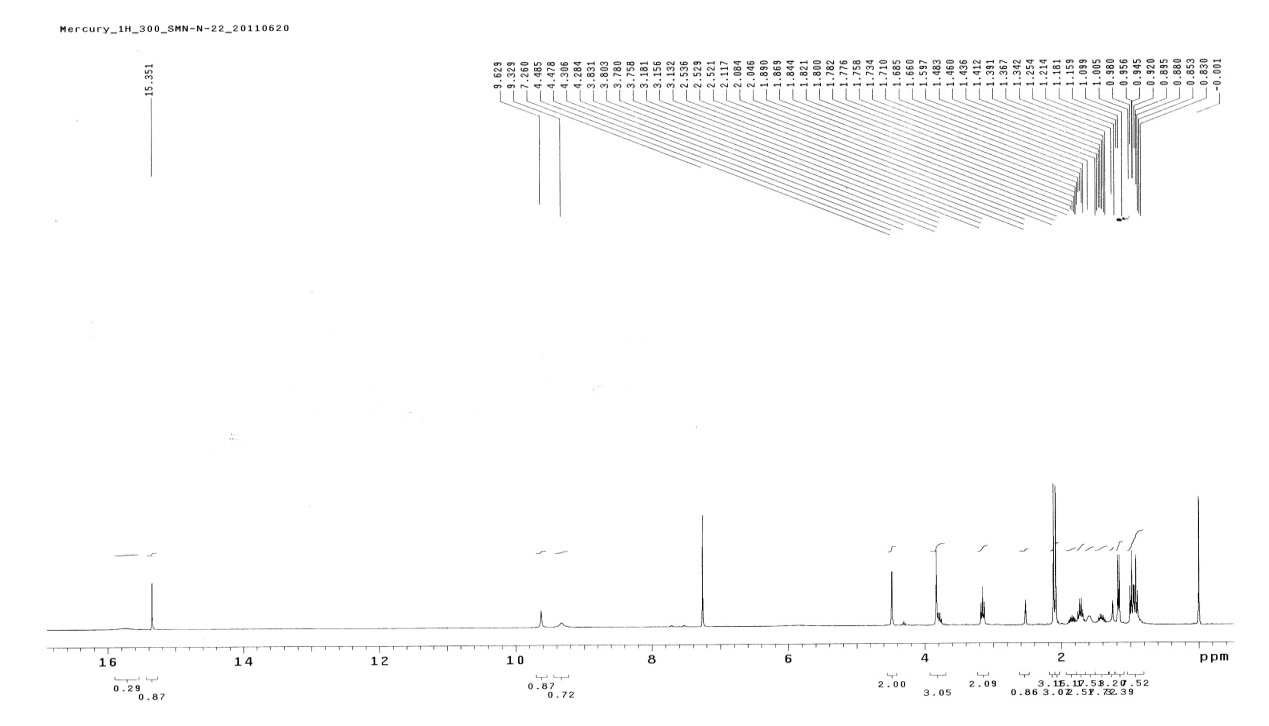


**S4 Fig.** **^13^C NMR (150 MHz, CDCl_3_) of a1**


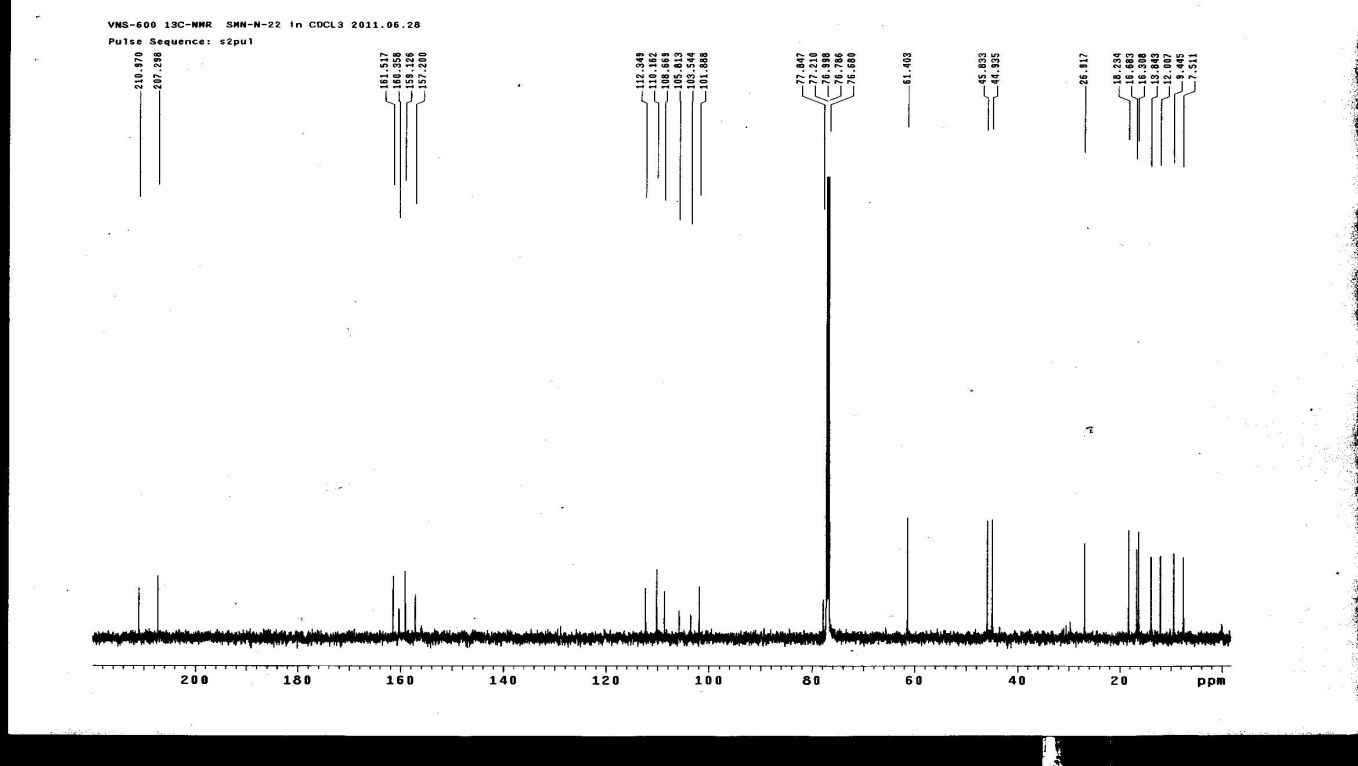


**S5 Fig. HRMS of a1**

**S6 Fig. ^1^H NMR (300 MHz, CDCl_3_) of a2**


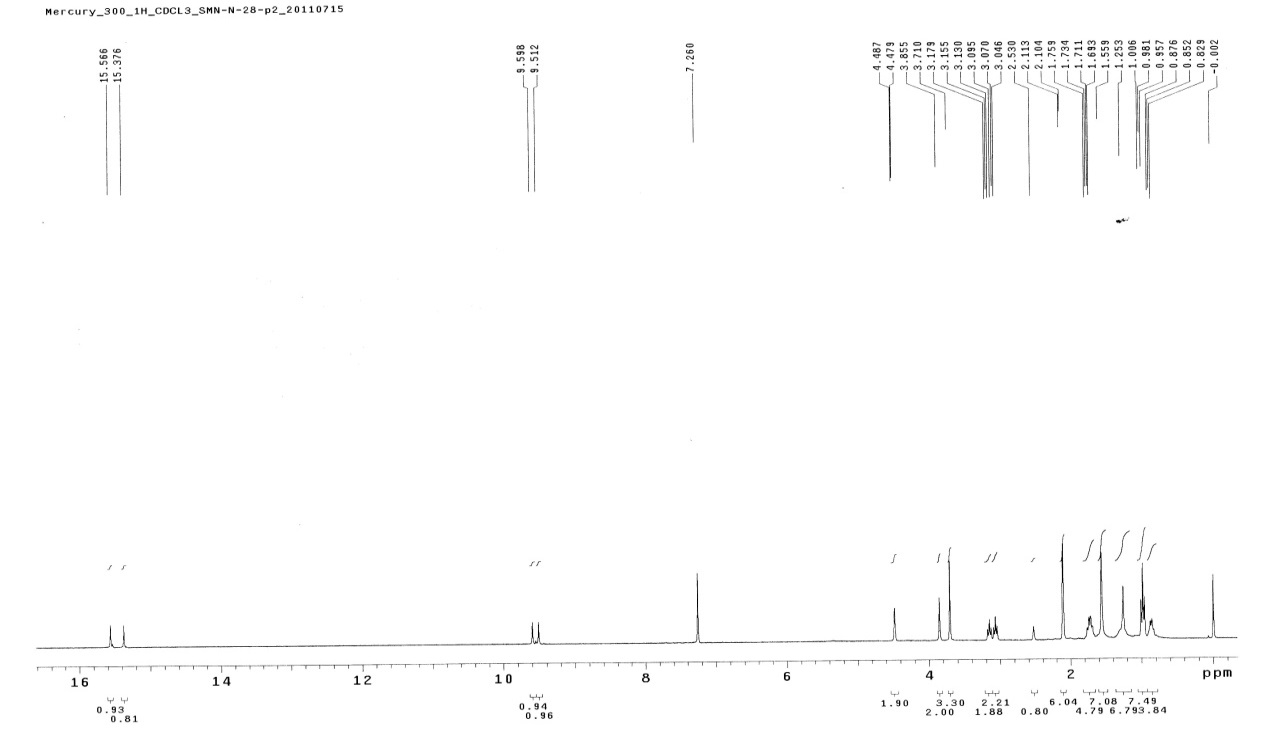


**S7 Fig. ^13^C NMR (75 MHz, CDCl_3_) of a2**


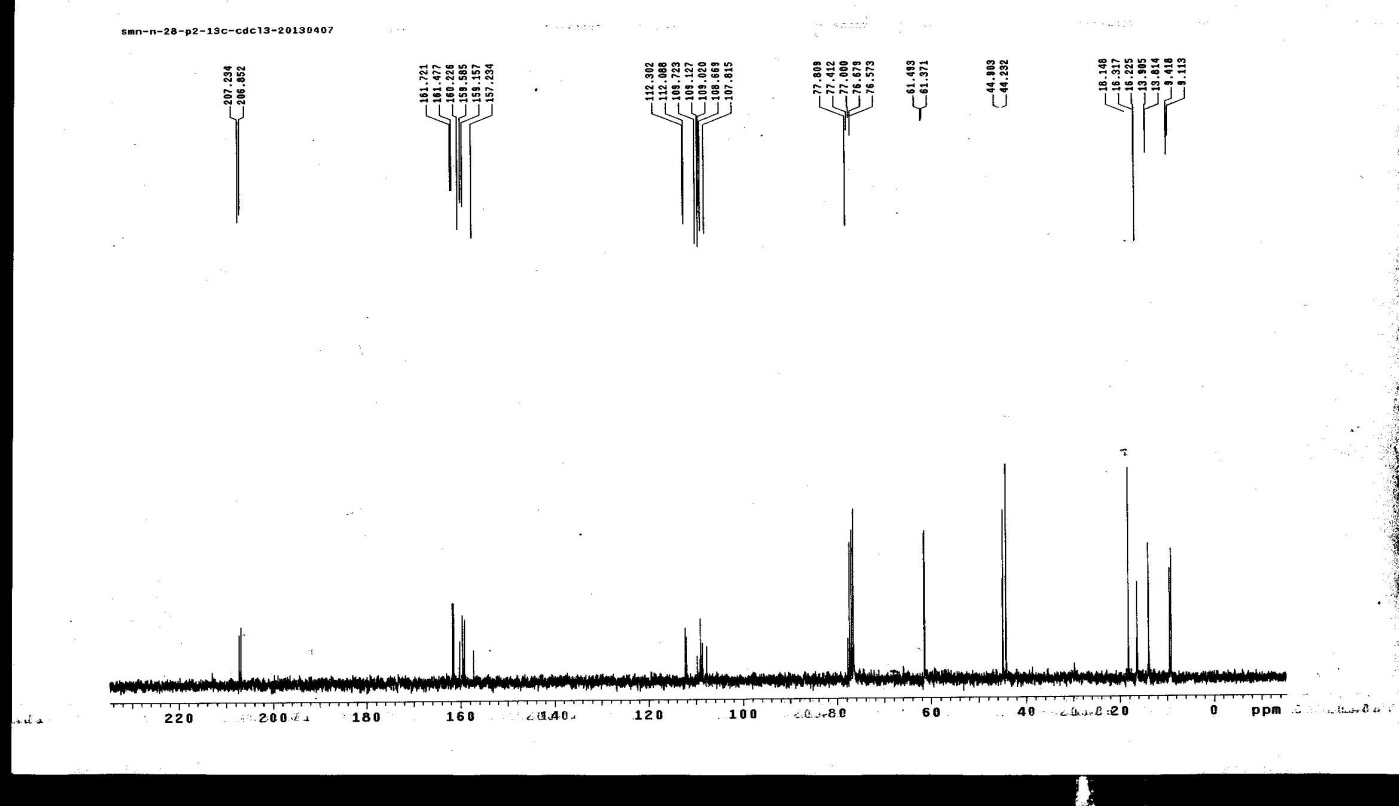


**S8 Fig. HRMS of a2**

**S9 Fig. ^1^H NMR (300 MHz, DMSO*-d_6_*) of a1b**


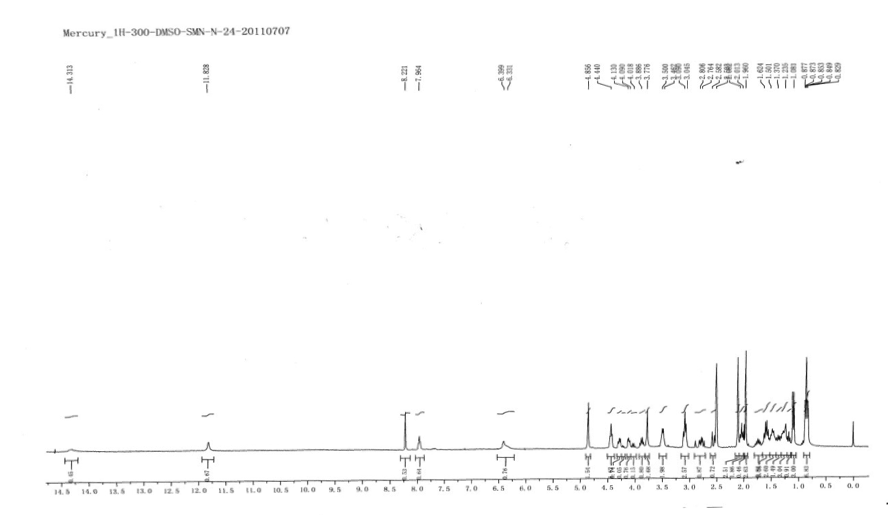


**S10 Fig. ^13^C NMR (125 MHz, DMSO*-d_6_*) of a1b**


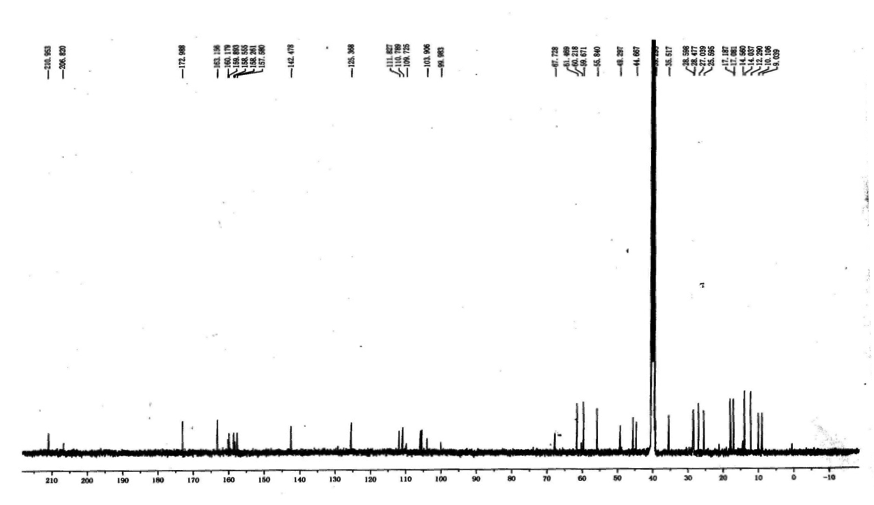


**S11 Fig. HRMS of a1b**

**S12 Fig. ^1^H NMR (300 MHz, DMSO*-d_6_*) of a2b**


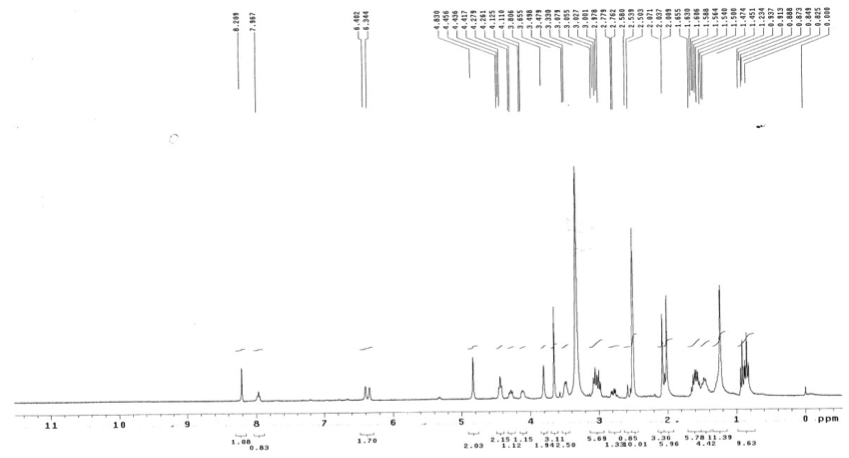


**S13 Fig. ^13^C NMR (125 MHz, DMSO*-d_6_*) of a2b**


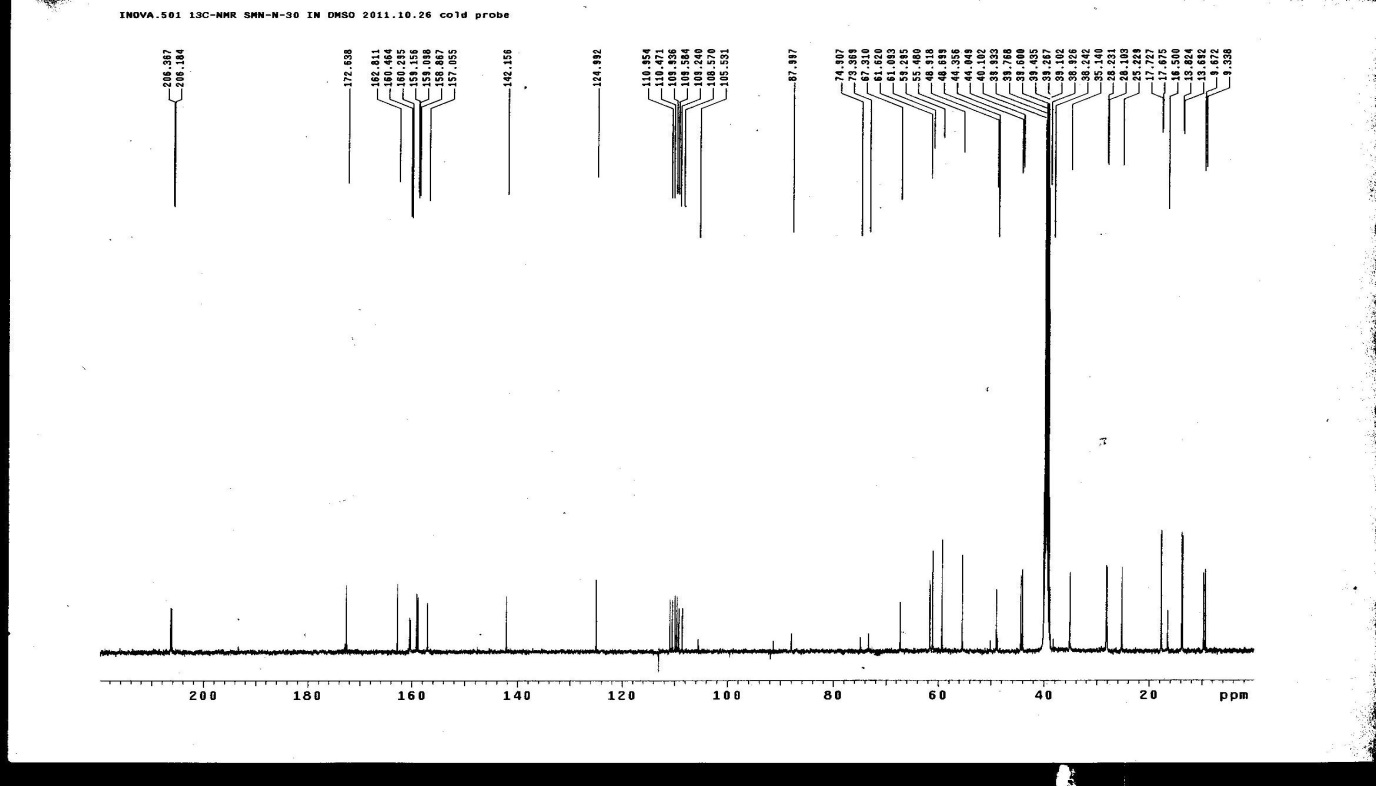


**S14 Fig. HRMS of a2b**

**Synthetic methods**

**Synthesis of 2,4,6-trihydroxybenzaldehyde (1)**

To the solution of DMF (1.16 mL, 15 mmol) in a round-bottom flask was added POCl_3_ (1.37 mL, 15 mmol) with stirring at 0 °C. The reaction mixture was stirred for 30 min at room temperature and then was dropwise added to the solution of starting material **0** (2.43 g, 15 mmol) in dioxane (6 mL) with strong stirring at 0 °C. The mixture was stirred at room temperature for 2 hours and then poured into ice-cold water [1]. The orange crystal formed was filtered and washed with ice-cold water to give 1.87 g of **1**. Crude yield was 81%. ^1^H NMR (300 MHz, DMSO-*d_6_*) δ 11.46 (s, 2H), 10.65 (s, 1H), 9.92 (s, 1H), 5.78 (s, 2H).

**Synthesis of 2-methylbenzene-1,3,5-triol (2)**

To a stirred solution of **1** (1.56 g, 10 mmol) in THF (200 mL) was added NaBH_3_CN (3.14 g, 50 mmol). The mixture was added 2 N HCl dropwise until there was no bubbles generated and then stirred at room temperature for 2 hours [2]. The precipitates were filtered off and the filtrate was concentrated in vacuum. Water (50 mL) was added to the residue followed by extraction with ethyl acetate (3 × 30 mL). The organic layers were combined and evaporated in vacuum. **2** was obtained as white powder (0.96 g) after purification by silica gel chromatography eluting with petroleum ether-ethyl acetate (5:1, v/v). Yield was 68%. ^1^H NMR (300 MHz, DMSO-*d_6_*) δ 8.80 (s, 2H), 8.67 (s, 1H), 5.75 (s, 2H), 1.79 (s, 3H).

**Synthesis of intermediates 1-(2,4,6-trihydroxy-3-methylphenyl)butan-1-one (3) and 2-methyl-1-(2,4,6-trihydroxy-3-methylphenyl)butan-1-one (4)**

To a stirred solution of AlCl_3_ (2.0 g, 15 mmol) in 1,2-dichloroethane (10 mL) was added Butyryl chloride (1.14 mL, 11 mmol) or 2-methylbutyryl chloride (1.36 mL, 11 mmol) and the mixture was stirred at room temperature for 10 min. After the addition of substrate **2** (1.40 g, mmol), the mixture was heated at 50 °C until the completion was detected by TLC. Then the hot reaction mixture was cooled to room temperature and poured into ice-cold water (50 mL) followed by extraction with ethyl acetate (3 × 30 mL). The organic layers were combined and evaporated in vacuum. The corresponding purified products **3** and **4** were obtained by silica gel chromatography in 85% and 83% yield, respectively. **3** was light yellow powder. ^1^H NMR (300 MHz, DMSO-*d_6_*) δ 14.02 (s, 1H), 10.49 (s, 1H), 10.25 (s, 1H), 6.00 (s, 1H), 2.97 (t, *J* = 7.2 Hz, 2H), 1.83 (s, 3H), 1.64 (m, 2H), 0.91 (t, *J* = 7.2 Hz, 3H). **4** was yellow powder. ^1^H NMR (300 MHz, DMSO-*d_6_*) δ 14.10 (s, 1H), 10.51 (s, 1H), 10.27 (s, 1H), 6.01 (s, 1H), 3.80 (m, 1H), 1.84 (s, 3H), 1.72 (m, 1H), 1.30 (m, 1H), 1.06 (d, *J* = 6.3 Hz, 3H), 0.84 (t, *J* = 7.2 Hz, 3H).

**Synthesis of 1-(2-(prop-2-yn-1-yloxy)3-methyl-4,6-dihydroxyphenyl)butan-1-one (8) and 1-(2-methoxy-3-methyll-4,6-dihydroxypheny)butan-1-one (9)**

To a stirred solution of **3** (1.51 g, 7.14 mmol) in acetone (70 mL) was added K_2_CO_3_ (7.40 g, 53.55 mmol) and MOMCl (1.36 mL, 17.85 mmol). The mixture was stirred at room temperature about 2 hours until the completion was detected by TLC. The insoluble substances were filtered off and the filtrate was evaporated in vacuum. **5** was obtained as white solid (1.89 g) after purification by silica gel chromatography eluting with petroleum ether-ethyl acetate (30:1, v/v). Crude yield was 89%. ^1^H NMR (300 MHz, DMSO-*d_6_*) δ 13.68 (s, 1H), 6.38 (s, 1H), 5.29 (s, 2H), 5.27 (s, 2H), 3.44 (s, 3H), 3.39 (s, 3H), 3.00 (t, *J* = 7.2 Hz, 2H), 1.94 (s, 3H), 1.62 (m, 2H), 0.92 (t, *J* = 7.2 Hz, 3H).

To a stirred solution of **5** (1.49 g, 5.0 mmol) in acetone (20 mL) was added K_2_CO_3_ (2.07 g, 15.0 mmol). Five minutes later, the reaction mixture was added Me_2_SO_4_ (1.42 mL, 15.0 mmol) and then refluxed for about 4 hours until the completion was detected by TLC. The insoluble substances were filtered off and the filtrate was evaporated in vacuum. **6** was obtained as colorless oil (1.28 g) after purification by silica gel chromatography eluting with petroleum ether-ethyl acetate (30:1, v/v). Crude yield was 82%; ^1^H NMR (300 MHz, DMSO-*d_6_*) δ 6.67 (s, 1H), 5.21 (s, 2H), 5.13 (s, 2H), 3.60 (s, 3H), 3.39 (s, 3H), 3.34 (s, 3H), 2.67 (t, *J* = 7.2 Hz, 2H), 2.02 (s, 3H), 1.58 (m, 2H), 0.90 (t, *J* = 7.2 Hz, 3H). To a stirred solution of **6** (0.94 g, 3 mmol) in MeOH (5 mL) was dropwise added concentrated HCl (0.5 mL). The mixture was refluxed for about 30 minutes until the completion was detected by TLC. **9** was obtained as light yellow solid (0.63 g) after purification by silica gel chromatography eluting with petroleum ether-ethyl acetate (20:1, v/v). Yield was 93%; ^1^H NMR (300 MHz, DMSO-*d_6_*) δ 12.30 (s, 1H), 10.35 (s, 1H), 6.15 (s, 1H), 3.65 (s, 3H), 2.92 (t, *J* = 7.2 Hz, 2H), 1.94 (s, 3H), 1.58 (m, 2H), 0.89 (t, *J* = 7.2 Hz, 3H).

To a stirred solution of **5** (0.30 g, 1.0 mmol), propargyl alcohol (0.17 mL, 3.0 mmol) and PPh_3_ (0.66 g, 2.5 mmol) in THF (5 mL) was dropwise added DEAD (0.39 mL, 2.5 mmol). The mixture was stirred at room temperature for about 30 min until the completion was detected by TLC. **7** was obtained as colorless oil after purification by silica gel chromatography eluting with petroleum ether-ethyl acetate (30:1, v/v). Yield was 66%; ^1^H NMR (300 MHz, Acetone-*d_6_*) δ 6.80 (s, 1H), 5.24 (s, 2H), 5.17 (s, 2H), 4.52 (d, *J* = 2.4 Hz, 2H), 3.46 (s, 1H), 3.45 (s, 3H), 3.42 (s, 3H), 2.75 (t, *J* = 7.2 Hz, 2H), 2.13 (s, 3H), 1.65 (m, 2H), 0.95 (t, *J* = 7.2 Hz, 3H). **8** was prepared from **7** in a similar manner to the synthesis of **9** from **6** as off white solid. Yield 83%; ^1^H NMR (300 MHz, Acetone-*d_6_*) δ 13.10 (s, 1H), 9.46 (s, 1H), 6.24 (s, 1H), 4.65 (d, *J* = 2.4 Hz, 2H), 3.14 (m, 3H), 2.10 (s, 3H), 1.70 (m, 2H), 0.95 (t, *J* = 7.5 Hz, 3H).

**Synthesis of 1-(3-(3-butyryl-2,6-dihydroxy-5-methyl-4-(prop-2-yn-1-yloxy)benzyl)-2,4,6-trihydroxy-5-methylphenyl)-2-methylbutan-1-one (a1)**

To a solution of intermediates **4** (23 mg, 0.1 mmol) and **8** (25 mg, 0.1 mmol) in acetic acid (1 mL) was added 12 droplets HCHO solution (37%-40%) [3]. The mixture was stirred at room temperature for about 8 hours and then was added with ethyl acetate (5 mL). The solution was neutralized with saturated NaHCO_3_ solution until there were no bubbles generated. The organic layer was evaporated in vacuum. **a1** was obtained as light yellow solid (28 mg) after purification by silica gel column chromatography eluting with petroleum ether-ethyl acetate (40:1, v/v). Yield was 58%. Melting point is 93-95 °C. ^1^H NMR (300 MHz, CDCl_3_) δ 15.72 (s, 1H), 15.35 (s, 1H), 9.63 (s, 1H), 9.33 (s, 1H), 4.48 (d, *J* = 2.1 Hz, 2H), 3.83 (s, 2H), 3.77 (m, 1H), 3.16 (t, *J* = 7.5 Hz, 2H), 2.53 (t, *J* = 2.1 Hz, 1H), 2.12 (s, 3H), 2.08 (s, 3H), 1.83 (m, 1H), 1.72 (m, 2H), 1.40 (m, 1H), 1.17 (d, *J* = 6.6 Hz, 3H), 0.96 (t, *J* = 7.5 Hz, 3H), 0.85 (t, *J* = 7.5 Hz, 3H). ^13^C NMR (150 MHz, CDCl_3_) δ 211.0, 207.3, 161.7, 161.5, 160.3, 159.1, 159.0, 157.2, 112.4, 110.2, 108.7, 105.8, 103.5, 101.9, 77.8, 76.7, 61.4, 45.8, 44.9, 26.9, 18.2, 16.7, 16.3, 13.8, 12.0, 9.5, 7.5. HRMS 485.21699 was calculated for [M+H]^+^ and 485.21707 was observed. The molecular formular is C_27_H_32_O_8_.

**Synthesis of 1-(3-(3-butyryl-2,6-dihydroxy-4-methoxy-5-methylbenzyl)-2,4-dihydroxy-5-methyl-6-(prop-2-yn-1-yloxy)phenyl)butan-1-one (a2)**

**a2** was prepared from intermediates **8** and **9** in a similar manner to the synthesis of **a1** as light yellow solid. Yield was 41%; Melting point is 98-100 °C. ^1^H NMR (300 MHz, CDCl_3_) δ 15.57 (s, 1H), 15.38 (s, 1H), 9.60 (s, 1H), 9.51 (s, 1H), 4.48 (d, *J* = 2.4 Hz, 2H), 3.86 (s, 2H), 3.71 (s, 3H), 3.16 (t, *J* = 7.2 Hz, 2H), 3.07 (t, *J* = 7.2 Hz, 2H), 2.53 (t, *J* = 2.4 Hz, 1H), 2.11 (s, 3H), 2.10 (s, 3H), 1.73 (m, 2H), 1.25 (m, 2H), 0.98 (t, *J* = 7.2 Hz, 3H), 0.85 (t, *J* = 7.2 Hz, 3H). ^13^C NMR (75 MHz, CDCl_3_) δ 207.2, 206.9, 161.7, 161.5, 160.2, 159.6, 159.2, 157.2, 112.3, 112.1, 109.7, 109.0, 108.7, 107.8, 77.8, 76.6, 61.5, 61.4, 44.9, 44.2, 18.1, 16.3, 16.2, 13.9, 13.8, 9.4, 9.1. HRMS 485.21699 was calculated for [M+H]^+^ and 485.21677 was observed. The molecular formular is C_27_H_32_O_8_.

**Synthesis of a1b**

To a solution of 2-bromoethylamine hydrobromide (1.02 g, 5.0 mmol) in water (10 mL) was slowly added NaN_3_ (0.98 g, 15 mmol). The resulting solution was allowed to stir at reflux overnight. After cooling to room temperature, about 2/3 of the water was removed by evaporation and the remaining residue diluted with ether (20 mL). This biphasic mixture was cooled to 0 °C and KOH pellets (1.3 g) was slowly added. The phases separated and the aqueous phase extracted with ether (2 × 15 mL). All organics combined, dried over Na_2_SO_4_, and concentrated to afford 2-azidoethylamine as colorless oil in 70% yield [4]. To a solution of biotin (488 mg, 2.0 mmol) and HOSu (246 mg, 2.14 mmol) in DMF (20 mL) was added EDC^.^HCl (431 mg, 2.25 mmol) and the reaction allowed to sitr at room temperature overnight. The reaction was concentrated to dryness and the residue was washed with MeOH (2 × 2 mL) to afford biotin-OSu as white solid in 89% yield. To a solution of biotin-OSu (341 mg, 1.0 mmol) and TEA (832 μL, 6.0 mmol) in DMF (6 mL) was added 2-azidoethylamine (129 mg, 1.5 mmol) and the reaction allowed to sitr at room temperature overnight. The reaction was concentrated to dryness and the residue was purified by silica gel chromatography eluting with DCM-MeOH (20:1, v/v) to afford biotinylated azide as white solid. Yield was 84%. ^1^H NMR (300 MHz, DMSO*-d_6_*) δ 8.02 (t, *J* = 5.8 Hz, 1H), 6.40 (s, 1H), 6.34 (s, 1H), 4.30 (m, 1H), 4.10 (m, 1H), 3.41 (m, 1H), 3.19 (m, 2H), 3.09 (m, 2H), 2.81 (dd, *J* = 12.4, 5.0 Hz, 1H), 2.56 (d, *J* = 12.4 Hz, 1H), 2.06 (t, *J* = 7.2 Hz, 2H), 1.45 (m, 6H).

To a suspension of **a1** (0.1 mmol) and biotinylated azide (0.1 mmol) in a 1:1 mixture of *t*-BuOH and water (1.0 mL) were added a freshly prepared 1.0 M solution of sodium ascorbate (0.2 mL, 0.2 mmol) in water, followed by the addition of 0.1 M solution of copper sulfate pentahydrate in water (0.1 mL, 0.01 mmol) ^[5]^. The reaction mixture was stirred at 45 °C for about 12 hours. After completion monitored by TLC, the reaction mixture was evaporated. **a1b** was obtained as light yellow solid (24 mg) after purification by silica gel chromatography eluting with DCM-MeOH (10:1, v/v). Yield was 30%. Melting point is 148-150 °C. ^1^H NMR (300 MHz, DMSO*-d_6_*) δ 14.31 (s, 1H), 11.82 (s, 1H), 8.22 (s, 1H), 7.96 (m, 1H), 6.40 (s, 1H), 6.33 (s, 1H), 4.86 (s, 2H), 4.40 (t, *J* = 6.0 Hz, 2H), 4.20 (m, 1H), 4.09 (m, 1H), 3.89 (m, 1H), 3.77 (s, 2H), 3.46 (t, *J* = 6.0 Hz, 2H), 3.09 (m, 1H), 3.05 (m, 2H), 2.78 (dd, *J* = 12.0, 4.8 Hz, 1H), 2.58 (d, *J* = 12.5 Hz, 1H), 2.10 (s, 3H), 2.04 (t, *J* = 7.5 Hz, 2H), 1.96 (s, 3H), 1.82 (m, 1H), 1.62 (m, 2H), 1.40 (m, 1H), 1.37 (m, 2H), 1.24 (m, 4H), 1.09 (d, *J* = 6.9 Hz, 3H), 0.87 (m, 3H), 0.82 (m, 3H). ^13^C NMR (125 MHz, DMSO*-d_6_*) δ 207.0, 206.8, 173.0, 163.2, 160.5, 160.2, 159.9, 158.6, 158.3, 157.6, 142.5, 125.4, 111.8, 110.8, 109.7, 105.3, 104.9, 99.9, 67.7, 61.5, 59.7, 55.8, 49.3, 48.7, 45.7, 44.7, 38.9, 35.5, 28.6, 28.5, 27.0, 25.6, 17.2, 17.1, 14.6, 14.0, 12.3, 10.1, 9.0. HRMS 797.35384 was calculated for [M+H]^+^ and 797.35455 was observed. The molecular formular is C_39_H_52_O_10_N_6_S.

**Synthesis of a2b**

**a2b** was prepared from **a2** and biotinylated azide in a similar manner to the synthesis of **a1b** as white solid. Yield is 40%. Melting point is 148-149 °C. ^1^H NMR (300 MHz, DMSO*-d_6_*) δ 8.21 (s, 1H), 7.97 (m, 1H), 6.40 (s, 1H), 6.34 (s, 1H), 4.83 (s, 2H), 4.44 (t, *J* = 6.0 Hz, 2H), 4.22 (m, 1H), 4.11 (m, 1H), 3.81 (s, 2H), 3.66 (s, 3H), 3.45 (t, *J* = 6.0 Hz, 2H), 3.08 (m, 1H), 3.03 (m, 4H), 2.78 (dd, *J* = 12.1, 4.7 Hz, 1H), 2.58 (d, *J* = 12.5 Hz, 1H), 2.07 (s, 3H), 2.04 (m, 2H), 2.01 (s, 3H), 1.63 (m, 2H), 1.23 (m, 2H), 1.45 (m, 6H), 0.91 (t, *J* = 7.2 Hz, 3H), 0.85 (t, *J* = 7.2 Hz, 3H). ^13^C NMR (125 MHz, DMSO*-d_6_*) δ 206.4, 206.2, 172.6, 162.8, 160.5, 160.3, 159.2, 159.1, 158.9, 157.1, 142.2, 125.0, 110.9, 110.5, 109.9, 109.6, 109.2, 108.6, 67.3, 61.6, 61.1, 59.3, 55.5, 48.9, 48.7, 44.4, 44.1, 38.9, 35.1, 28.2, 28.1, 25.2, 17.7, 17.7, 16.5, 13.8, 13.7, 9.7, 9.3. HRMS 797.35384 was calculated for [M+H]^+^ and 797.35406 was observed. The molecular formular is C_39_H_52_O_10_N_6_S.

**References**

1. Bharate SB, Bhutani KK, Khan SI, Tekwani BL, Jacob MR, Khan IA, et al. (2006) Biomimetic synthesis, antimicrobial, antileishmanial and antimalarial activities of euglobals and their analogues. Bioorg Med Chem 14(6): 1750-1760.
2. Elliger CA. (1985) Deoxygenation of aldehydes and ketones with sodium cyanoborohydride. Synthesis Communications 15(14): 1315-1324.
3. Yao RH, Ma RS, Chen YQ, Huang LS. (1984) Synthesis of analogs of agrimophol. Yao Xue Xue Bao 19(3): 228-231.
4. Lampkins AJ, O'Neil EJ, Smith BD. (2008) Bio-orthogonal phosphatidylserine conjugates for delivery and imaging applications. J Org Chem 73(16): 6053-6058.

Rostovtsev VV, Green LG, Fokin VV, Sharpless KB. (2002) A stepwise huisgen cycloaddition process: copper(I)-catalyzed regioselective "ligation" of azides and terminal alkynes. Angew Chem Int Ed Engl 41(14): 2596-2599.
